# Venous thromboembolism and secondary outcomes of bleeding and mortality in patients with gliomas: a multicenter cohort study

**DOI:** 10.3389/fonc.2026.1771694

**Published:** 2026-05-21

**Authors:** Viviane Cordeiro Veiga, Camilla Akemi Felizardo Yamada, Carlos Afonso Clara, Jessica Carolina Andrade dos Santos, Breno Gray Milano, Danielli de Almeida Matias, Pedro Hortêncio Saboia da Escossia Melo, Gabriel N. de Rezende Batistella, Alexandre Biasi Cavalcanti, Fabiana Spillari Viola, Ana Carolina Levy, Silvana Soares dos Santos, Carolina Fittipaldi, Mauro Bráulio da Rosa Pinto, Daniela Galvão Barros de Oliveira, Thiago Santos Vieira, Flavia Regina de Moraes, Thatiane Lopes Valentim Di Paschoaele Ostolin, Talita Rantin Belucci, Feres Chaddad-Neto, Alex Machado Baeta, Iuri Santana Neville, Stela Verzinhasse Peres

**Affiliations:** 1 BP – A Beneficência Portuguesa de São Paulo, São Paulo, Brazil; 2 Latin American Cooperative Oncology Group - Neuro, Society for Neuro-Oncology Latin America (SNOLA), São Paulo, Brazil; 3 Hospital de Amor - Fundação Pio XII, Barretos, São Paulo, Brazil; 4 ICESP - Instituto do Câncer de São Paulo, São Paulo, Brazil; 5 Department of Oncology, Liga Norte Riograndense Contra o Câncer, Natal, Brazil; 6 Institute of Education, Research, and Innovation, Liga Norte Riograndense Contra o Câncer, Natal, Brazil; 7 Hcor Research Institute, São Paulo, Brazil; 8 Hospital São Lucas da Pontifícia Universidade Católica, Rio Grande do Sul, Brazil; 9 AC Camargo Cancer Center, São Paulo, Brazil; 10 INCA – Instituto Nacional do Câncer, Rio de Janeiro, Brazil; 11 Hospital Santa Izabel - Santa Casa da Bahia, Salvador, Bahia, Brazil; 12 Department of Neurosurgery, BP - A Beneficência Portuguesa de São Paulo, São Paulo, Brazil; 13 Department of Neurology and Neurosurgery, Universidade Federal de São Paulo, São Paulo, Brazil; 14 Department of Neurology, BP – A Beneficência Portuguesa de São Paulo, São Paulo, Brazil

**Keywords:** central nervous system neoplasms, neoplasm complications, prognostic factors, upper-middle income countries, venous thromboembolism

## Abstract

**Introduction:**

Venous thromboembolism (VTE) is a common and clinically significant complication in Central Nervous system (CNS) tumors, associated with increased hospitalization, morbidity, and mortality. This study estimates the cumulative incidence of VTE, bleeding, and all-cause mortality in patients with primary gliomas and evaluates their determinants using multivariable models accounting for competing risks, confounding, and missing data.

**Methods:**

This multicenter retrospective cohort study evaluated 334 adults with histologically confirmed primary gliomas treated across nine Brazilian centers (2021–2022; follow-up to December 2023). Outcomes included VTE, bleeding, and all-cause mortality. Sociodemographic, clinical, tumor-specific, treatment, and thromboprophylaxis data were collected. Twelve-month cumulative incidence was estimated using competing-risk methods. VTE and bleeding were assessed with Fine–Gray sub distribution hazard models, and mortality with Cox proportional hazards models.

**Results:**

VTE and bleeding cumulative incidences at 12 months were 11.8% (8.0 – 15.5% CI) and 4.7% (2.1 – 7.3% CI). The cumulative mortality incidence was 26.2%. Prior VTE (SHRadj = 4.28; 95% CI 2.60 – 7.07) and corticosteroid use (SHRadj = 3.25; 95% CI 2.20 – 4.81) independently increased VTE risk. Chronic kidney failure (SHRadj = 6.45; 95% CI 5.39 – 7.73) and diabetes mellitus SHRadj = 2.11; 95% CI: 1.51 – 2.95) were the independent predictor of bleeding. For mortality, gliomas NOS (HRadj = 9.20; 95% CI: 5.38–15.73) and GBM (HRadj = 10.34; 95% CI 4.28 – 24.96) were strongly associated with increased risk. Longer hospitalization (≥7 days; HRadj = 2.01; 95% CI 1.81 – 2.24), postoperative bleeding (HRadj = 2.92; 95% CI 2.10 – 4.13), and treatment in specialized cancer centers (HRadj = 2.59; 95% CI: 1.50 – 4.50), along with demographic characteristics, were also associated with higher mortality.

**Conclusion:**

In this multicenter Brazilian cohort of patients with primary gliomas, VTE was frequent, while bleeding was less common but clinically relevant. Mortality risk was driven by patient characteristics, tumor aggressiveness, and hospitalization factors, underscoring the need for individualized risk stratification. Within the subgroup analysis for mortality, higher educational level was associated with lower hazard of death, whereas prolonged hospitalization and postoperative bleeding consistently increased mortality risk across both GBM and grade 3 or 4 tumors.

## 1 Introduction

Venous thromboembolism (VTE), which includes deep vein thrombosis (DVT) and pulmonary embolism (PE), is a well-recognized and potentially life-threatening complication among patients with central nervous system (CNS) tumors, contributing substantially to morbidity, mortality, and prolonged hospitalization (1–3). The incidence of symptomatic VTE in patients with glioblastoma (GBM) and high-grade gliomas (HGG) varies widely, ranging from 7% to 30%, and may reach cumulative incidences up to 60% when asymptomatic events are included (2, 4–8). The highest risk occurs in the first three months after diagnosis and persists for up to 24 months and throughout the disease course (2, 7, 8).

Multiple clinical and tumor-related factors contribute to VTE risk, including advanced age, male sex, poor performance status, prior VTE, prolonged surgery, increased intraoperative blood loss, high tumor grade, IDH-wildtype status, EGFR amplification, larger tumor volume, extended ICU stay, and concomitant long-course radiotherapy (7–12). Conversely, IDH mutations, 1p/19q codeletion, MGMT promoter methylation, and oligodendroglioma histology are associated with reduced VTE risk (5, 10, 13, 14). Regarding prophylaxis, anticoagulation to CNS tumors remains challenging due to the need to balance thrombotic and hemorrhagic risks. In gliomas, therapeutic anticoagulation is associated with a nearly four-fold increase in intracranial hemorrhage (ICH), particularly in primary tumors, with a lower risk in brain metastases (10, 15–17).

In this context, VTE remains a critical determinant of morbidity and mortality in CNS tumors. But aggressive anticoagulation strategies may substantially increase the risk of bleeding, which can independently worsen survival. The interplay between these complications creates a complex therapeutic challenge, where preventing thrombotic events must be carefully balanced against minimizing hemorrhagic risks. Assessing VTE, bleeding, and mortality concurrently provides a clinically meaningful framework for guiding thromboprophylaxis and anticoagulation strategies. Given the clinical impact of VTE in patients with gliomas, advancing precision risk stratification and safer prophylactic regimens are a priority for improving care in this high-risk population. Accordingly, this study aims to estimate the cumulative incidence of VTE, bleeding, and all-cause mortality in patients with primary gliomas and to investigate the clinical and biological determinants of these outcomes. Secondary analyses will further test the consistency of these findings using multivariable models addressing competing risks, confounding, and missing data.

## 2 Materials and methods

### 2.1 Study design and patients

This multicenter retrospective cohort study was supported by the *Programa de Apoio ao Desenvolvimento Institucional do Sistema Único de Saúde* (PROADI-SUS) and coordinated by BP – A Beneficência Portuguesa de São Paulo Hospital (Protocol No. NUP25000.1121542022-98). The study included patients across nine centers located in three Brazilian regions (Southeast, South, and Northeast), diagnosed from January 13, 2021, to December 31, 2022, and followed up until December 31, 2023 (**Supplementary Table 1**). The study was approved by the Research Ethics Committee of BP – A Beneficência Portuguesa de São Paulo (CAAE 64435822.6.1001.5483), as well as by the ethics committees of all participating centers. All procedures were conducted in accordance with the principles of the Declaration of Helsinki, and written informed consent was obtained from all participants.

#### 2.1.1 Inclusion and exclusion criteria

Patients aged ≥ 18 years with histologically confirmed primary CNS tumors, classified according to the fifth edition of the World Health Organization (WHO) Classification of Tumors of the Central Nervous System (18), were eligible. Exclusion criteria were metastatic tumors; CNS lymphomas; WHO grade 1 and 2 meningiomas; CNS tumors classified as grade 1 or cases with missing classification data; and COVID-19 diagnosis during the study period.

#### 2.1.2 Sample size

The sample size was calculated based on the primary outcome (i.e., VTE) assuming a significance level (α) of 5%, test power (1-β) of 80%, a hazard ratio (HR) of 1.8, and an estimated VTE incidence of 20% in the CNS tumor population (4). To ensure a conservative estimate of event non-occurrence, a minimum sample size of 320 participants was required. Participants were consecutively enrolled based on diagnosis data extracted from medical records.

Data was collected from 397 participants. After consistency checks, 63 patients were excluded: 37 due to missing tumor grade, 21 reclassified as WHO grade 1 after biopsy, and 5 with benign lesions. All classifications and reclassifications were independently reviewed and confirmed by three neuro-oncology specialists. The final sample comprised 334 eligible patients.

### 2.2 Outcomes

The primary outcome was VTE, including PE and DVT, defined as any symptomatic - patients commonly present with symptoms that prompt clinical investigation, including leg swelling, pain, or tenderness in cases of DVT, and chest pain, dyspnea, or cough in cases of PE - or incidental event involving the upper- or lower-limb events confirmed by imaging (venous Doppler ultrasound, chest computed tomography, pulmonary scintigraphy, or angiography). Arterial thromboembolic events and splenic vein thrombosis were excluded (2). The secondary outcomes included major bleeding, defined as any fatal or symptomatic hemorrhage in a critical site (intracranial, intraspinal, intraocular, retroperitoneal, pericardial spaces, non-operated joints, or intramuscular sites associated with compartment syndrome) (9) and all-cause mortality.

### 2.3 Covariates

Sociodemographic data extracted from medical records included sex, age group, and education level. Nutritional status was assessed using body mass index (BMI, kg/m²). Adults aged 18–60 years were classified according to the WHO (19) criteria, and older adults (> 60 years) according to Pan American Health Organization (PAHO) (20) guidelines. Alcohol consumption and smoking status were categorized as never or current/former drinker or smoker.

Comorbidity and prior medical history were recorded as binary variables (yes/no). Tumor type was categorized into four groups: 1. Oligodendrogliomas/Astrocytomas; 2. Gliomas not otherwise specified (NOS); 3. Glioblastomas (GBM); and 4. other tumor types (**Supplementary Table 2**). Additional tumor characteristics included histological grade (2 and 3/4); IDH wild-type (yes/no) (18); tumor location (infratentorial, intramedullary, or supratentorial); and tumor size (< 2.5 cm, 2.5 to 5 cm, and > 5 cm). Length of hospital stay (<7 days or ≥7 days) referred to the total duration of the initial hospitalization, from admission or surgery to discharge or death (14). Thromboprophylaxis was classified as none, pharmacological, mechanical, or combined, and duration was recorded as 1 month, 3 months, 6 months, during hospitalization, or other.

Postoperative variables (yes/no) included hemiparesis or hemiplegia, immobilization (inability to independently change position while lying down) (21), corticosteroid use, antiplatelet agents use, and adjuvant treatment (radiotherapy and/or chemotherapy). The laboratory variables, platelet count and International Normalized Ratio (INR), were described.

### 2.4 Statistical analysis

Data were captured in REDCap system and analyzed in RStudio (v4.2.3). Descriptive analyses were performed using absolute and relative frequencies, and measures of central tendency and dispersion. Cumulative incidence over 12 months was estimated with 95% confidence intervals (95% CI), considering death as competing risk. VTE and bleeding were analyzed using Fine–Gray models to obtain subdistribution hazard ratios (SHR) and all-cause mortality using Cox models to estimate hazard ratios (HR), both with 95% confidence intervals (CIs). Variables with a *p*-value < 0.20 in the univariate analysis were entered into multivariate models using stepwise approach (22). Potential confounding factors and interactions were assessed while independent variables remained in the multivariate model. To account for inter-institutional variability, cluster-robust standard errors were applied at the institutional level. Model adequacy was evaluated using the log-pseudolikelihood criterion. Missing values were reported in the descriptive tables. Sensitivity analyses were performed using the Multiple Imputation by Chained Equations (MICE) method, which imputes missing values using predictive models and combines results to account for imputation uncertainty. Inclusion criteria variables (sex, age, tumor type, and tumor grade) and outcome variables showed no missing values.

## 3 Results

### 3.1 Patient characteristics

Among the 334 patients included, 66.5% were male. The mean age at diagnosis was 51.7 years (SD = 15.2; range 18.9–82.1). The mean body mass index (BMI) was 27.2 kg/m² (SD = 5.0; range 16.8–56.3). Only three patients experienced both VTE and bleeding. Regarding educational level, 31.1% (n = 104) had completed higher education, followed by 23.4% (n = 78) who had completed high school. Approximately 20% of patients were current smokers and/or alcohol consumers. Hypertension was reported in 38.6% of the sample, and 4.8% had a prior history of VTE (**Supplementary Table 3**). GBM was the most frequent diagnosis (50.9%), and high-grade tumors (grades 3 and 4) accounted for 77.5% of cases (**Table 1**). Participants with gliomas who were eligible for chemotherapy, 13.2% developed VTE, 3.1% had bleeding events, and 25.3% died. Temozolomide was the most frequently administered chemotherapeutic agent, used in 92.2% of cases (**Supplementary Table 3**). Most thromboprophylaxis regimens were administered during hospitalization (**Supplementary Table 4**).

**Table 1:** Distribution of demographic, clinical, and laboratory characteristics among patients with gliomas (n=334).

Variables	Categories	n	(%)
VTE	No	293	(87.7)
	Yes	41	(12.3)
Bleeding	No	320	(95.8)
	Yes	14	(4.2)
Death	No	238	(71.3)
	Yes	96	(28.7)
Sex	Male	222	(66.5)
	Female	112	(33.5)
Age group	< 40	82	(24.6)
	40 - 60	144	(43.1)
	> 60	108	(32.3)
Education level	< Elementary school	56	(16.8)
	Elementary school	34	(10.2)
	High school	78	(23.4)
	Higher education	104	(31.1)
	Missing	62	(18.6)
Nutritional status	Underweight	25	(7.5)
	Eutrophic	110	(32.9)
	Overweight	105	(31.4)
	Obese	88	(26.3)
	Missing	6	(1.8)
Smoking	No	237	(71.0)
	Yes	65	(19.5)
	Missing	32	(9.6)
Alcoholism	No	230	(68.9)
	Yes	57	(17.1)
	Missing	47	(14.1)
Hypertension	No	202	(60.5)
	Yes	129	(38.6)
	Missing	3	(0.9)
Diabetes mellitus	No	271	(81.1)
	Yes	53	(15.9)
	Missing	10	(3.0)
Obesity	No	233	(69.8)
	Yes	79	(23.7)
	Missing	22	(6.6)
Chronic kidney failure	No	303	(90.7)
	Yes	2	(0.6)
	Missing	29	(8.7)
Congestive heart failure	No	304	4 (91)
	Yes	2	(0.6)
	Missing	28	(8.4)
Previous VTE	No	269	(80.5)
	Yes	16	(4.8)
	Missing	49	(14.7)
Variables	Categories	n	(%)
COPD	No	300	(89.8)
	Yes	9	(2.7)
	Missing	25	(7.5)
Previous myocardial infarction or stroke	No	295	(88.3)
	Yes	12	(3.6)
	Missing	27	(8.1)
Tumor type	Oligodendroglioma/ Astrocitoma	94	(28.1)
	Gliomas NOS*	44	(13.2)
	GBM	170	(50.9)
	Other	26	(7.8)
Tumor grade	2	75	(22.5)
	3 or 4	259	(77.5)
IDH wild-type	No	181	(54.2)
	Yes	107	7 (32)
	Missing	46	(13.8)
Local tumor	Infratentorial	16	(4.8)
	Intramedular	3	(0.9)
	Supratentorial	314	(94.0)
	Missing	1	(0.3)
Tumor size	< 2.5 cm	43	(12.9)
	2.5 to 5 cm	109	(32.6)
	≥ 5 cm	130	(38.9)
	Missing	52	(15.6)
Length of hospital days	< 7 days	140	(41.9)
	≥ 7 days	108	(32.3)
	Missing	86	(25.7)
Type of Prophylaxis	None	20	(6.0)
	Pharmacological	15	(4.5)
	Mechanical	85	(25.4)
	Combined	117	(35.0)
	Missing	97	(29.0)
Duration of prophylaxis	1 month	2	(0.6)
	3 months	2	(0.6)
	6 months	3	(0.9)
	During hospitalization	202	(60.5)
	Others	7	(2.1)
	Missing	118	(35.3)
Presence of hemiparesis/hemiplegia	No	195	(58.4)
	Yes	121	(36.2)
	Missing	18	(5.4)
Immobilization	No	239	(71.6)
	Yes	40	(12.0)
	Missing	55	(16.5)
Corticosteroid use	No	69	(20.7)
	Yes	249	(74.6)
	Missing	16	(4.8)
Antiplatelet use	No	249	(74.6)
	Yes	18	(5.4)
	Missing	67	(20.1)
Variables	Categories	n	(%)
Radiotherapy	No	54	(16.2)
	Yes	273	(81.7)
	Missing	7	(2.1)
Chemotherapy	No	70	(21.0)
	Yes	257	(76.9)
	Missing	7	(2.1)
Platelet count	n (median)	317 (230,000)	
	Min-Max	34,000 – 482,000	
	Missing	17	(5.1)
INR	n (median)	283 (1.0)	
	Min-Max	0.69 – 11.30	
	Missing	51	(15.3)

VTE, Venous Thromboembolisms; COPD, Chronic Obstructive Pulmonary Disease; Gliomas NOS, Gliomas not otherwise specified; GBM, Glioblastomas; INR, International Normalized Ratio.

**Figure 1:**
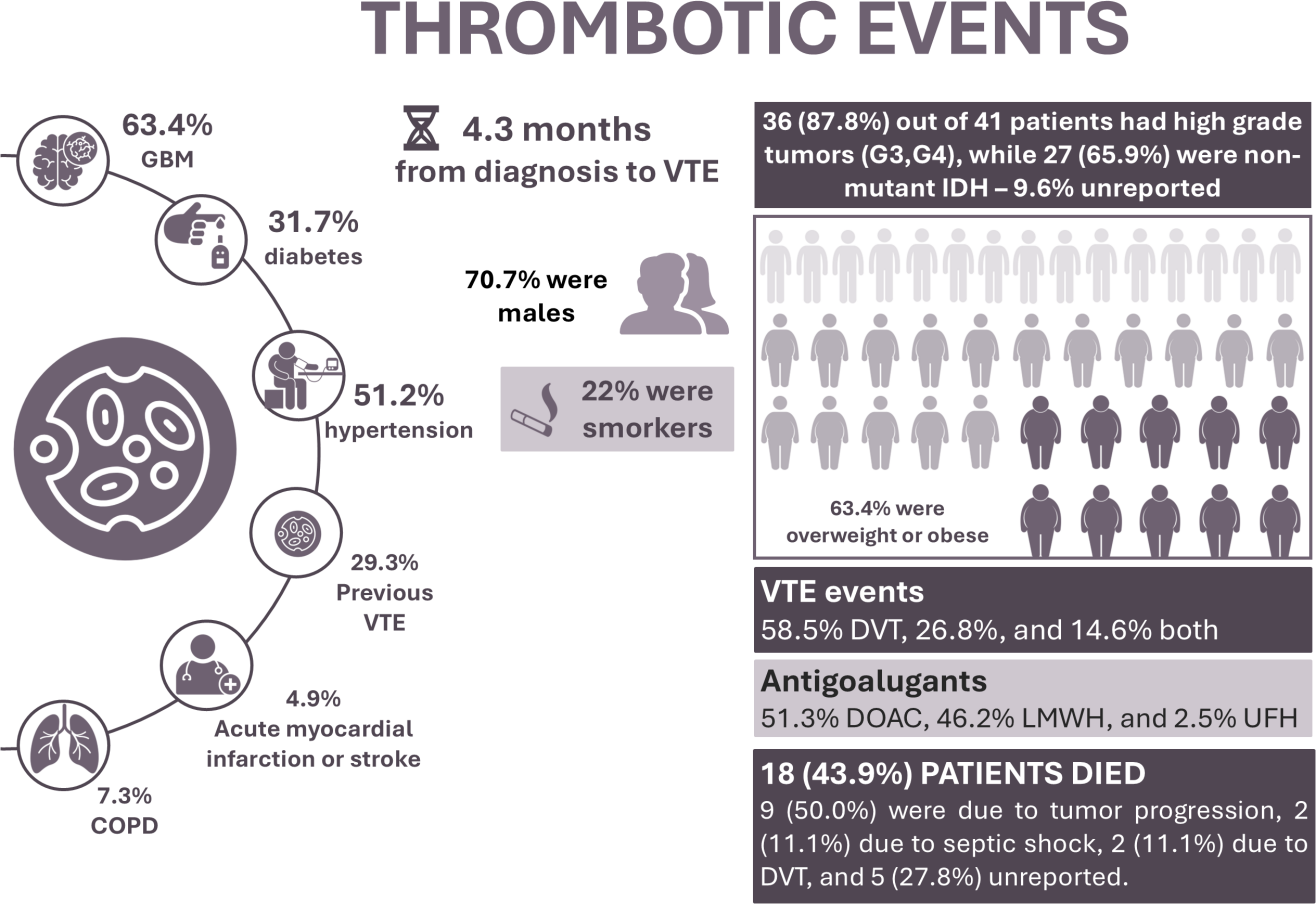
Characteristics of participants who developed VTE.

Among the 41 patients who developed VTE, 18 (43.9%) were identified during the hospitalization period. Regarding tumor characteristics, 26 had GBMs (63.4%) and 36 had high-grade tumors (grades 3 and 4, 87.8%). Non-mutant IDH status was identified in 27 patients (65.9%). Most were male (70.7%) and overweight/obese (63.4%). Only 17.1% (n = 7) were younger than 40 years. Hypertension was present in 51.2%, diabetes in 31.7%, smoking in 22%, Chronic Obstructive Pulmonary Disease (COPD) in 7.3%, and prior myocardial infarction or stroke in 4.9%. Twelve patients (29.3%) reported a history of prior VTE. No patients had documented thrombophilia, chronic kidney disease, or heart failure. Regarding thromboprophylaxis, 24.4% received combined prophylaxis and 22.0% mechanical prophylaxis only. Most patients underwent radiotherapy and chemotherapy (82.9%), predominantly with temozolomide. DVT was the most frequent event (58.5%), followed by PE (26.8%) and combined events (14.6%). Anticoagulation was administered to 95.1% of VTE patients, mainly direct oral anticoagulants (DOACs) (51.3%) and low – molecular – weight heparin (LMWH) (46.2%). Overall, 43.9% (n = 18) died, primarily due to disease progression (50%). Most deaths occurred in patients with GBM (77.8%), high-grade tumors (88.9%), IDH-wildtype status (88.2%), and hypertension (55.6%) (**Figure 1**). Detailed data are provided in **Supplementary Tables 4, 5**.

**Figure 2:**
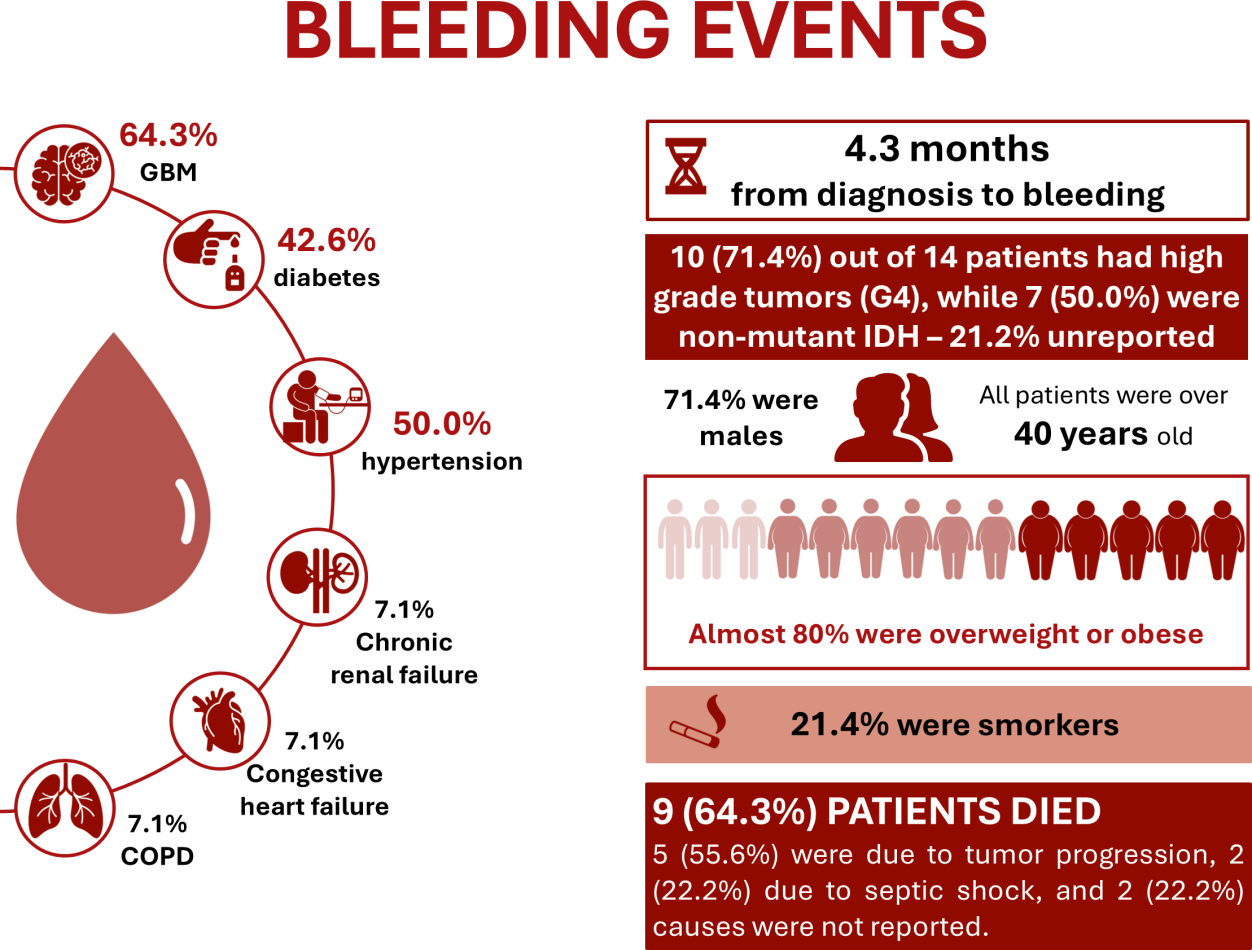
Characteristics of participants who developed bleeding.

Among the 14 patients with bleeding events, most had high-grade tumors (12 grade 3 and 4, including 10 grade 4), nine had GBMs, and seven had non-mutant IDH. The majority were male, over 40 years old, and overweight, with hypertension and diabetes as the most frequent comorbidities. No patient had a prior history of thrombosis, myocardial infarction, or hemorrhagic events. Among the 14 patients with bleeding events, seven received only mechanical postoperative prophylaxis, and nine had prophylaxis restricted to hospitalization. Eight underwent radiotherapy and chemotherapy, mainly with temozolomide. Bleeding occurred predominantly in the gliomas (n = 11). Seven patients required ICU admission, three needed transfusions, and three received preventive prophylaxis (one enoxaparin, one clexane, and one mechanical). Overall, nine patients died: five due to tumor progression, two from septic shock, and two with unreported causes. Of these deaths, eight occurred in patients with GBM or grade 3/4 tumors (88.9%), six had non-mutant IDH (66.7%), seven were male, and all three patients with a previous thrombotic event died (**Figure 2**). Detailed data are provided in **Supplementary Tables 4, 5**.

**Figure 3:**
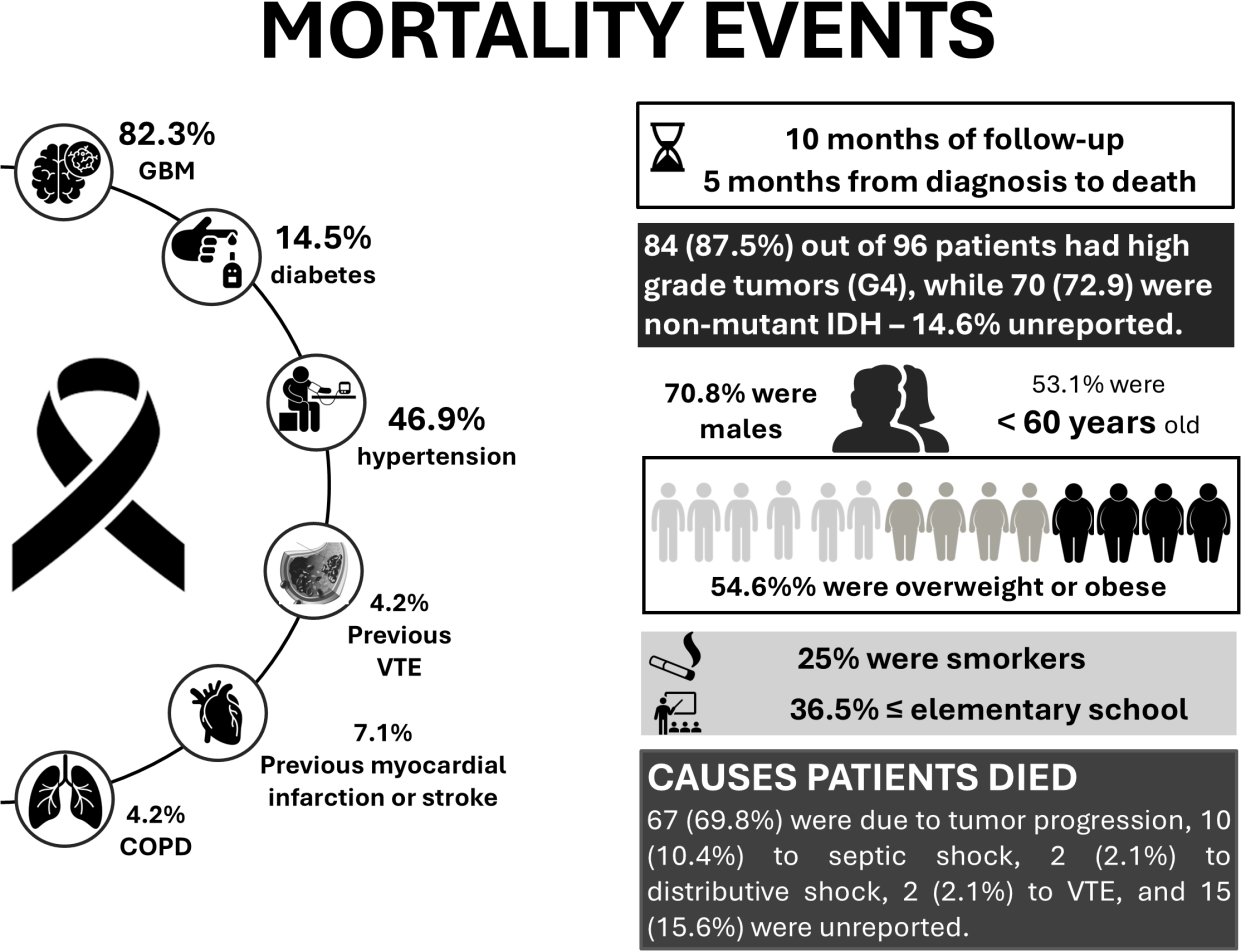
Characteristics of participants who died.

Among the patients who died during follow-up (n = 96), 53.1% were younger than 60 years, and 36.5% had completed elementary school or less. Most deaths occurred in patients with high-grade tumors, particularly GBM, which accounted for 82.3% of cases, and 70 patients (72.9%) had IDH wild-type tumors. Comorbidities were frequent, with hypertension reported in 46.9% and diabetes in 14.5% of patients. A prior history of venous thromboembolism was uncommon (4.2%), as were previous myocardial infarction or stroke (7.1%) and chronic obstructive pulmonary disease (4.2%) (**Figure 3**). Detailed data are provided in **Supplementary Tables 4, 5**.

Across subgroups, the median platelet count was 182,794 (range: 85,000–440,000; n = 39) for VTE, 180,000 (range: 75,000–462,000; n = 13) for bleeding, and 207,000 (range: 45,000–482,000; n = 94) for death. Regarding INR, the median value was 1.0 across all subgroups, with ranges of 0.75–10.60 (n = 37) for VTE, 0.86–1.10 (n = 12) for bleeding, and 0.75–10.60 (n = 87) for death (**Supplementary Table 5**).

**Figure 4:**
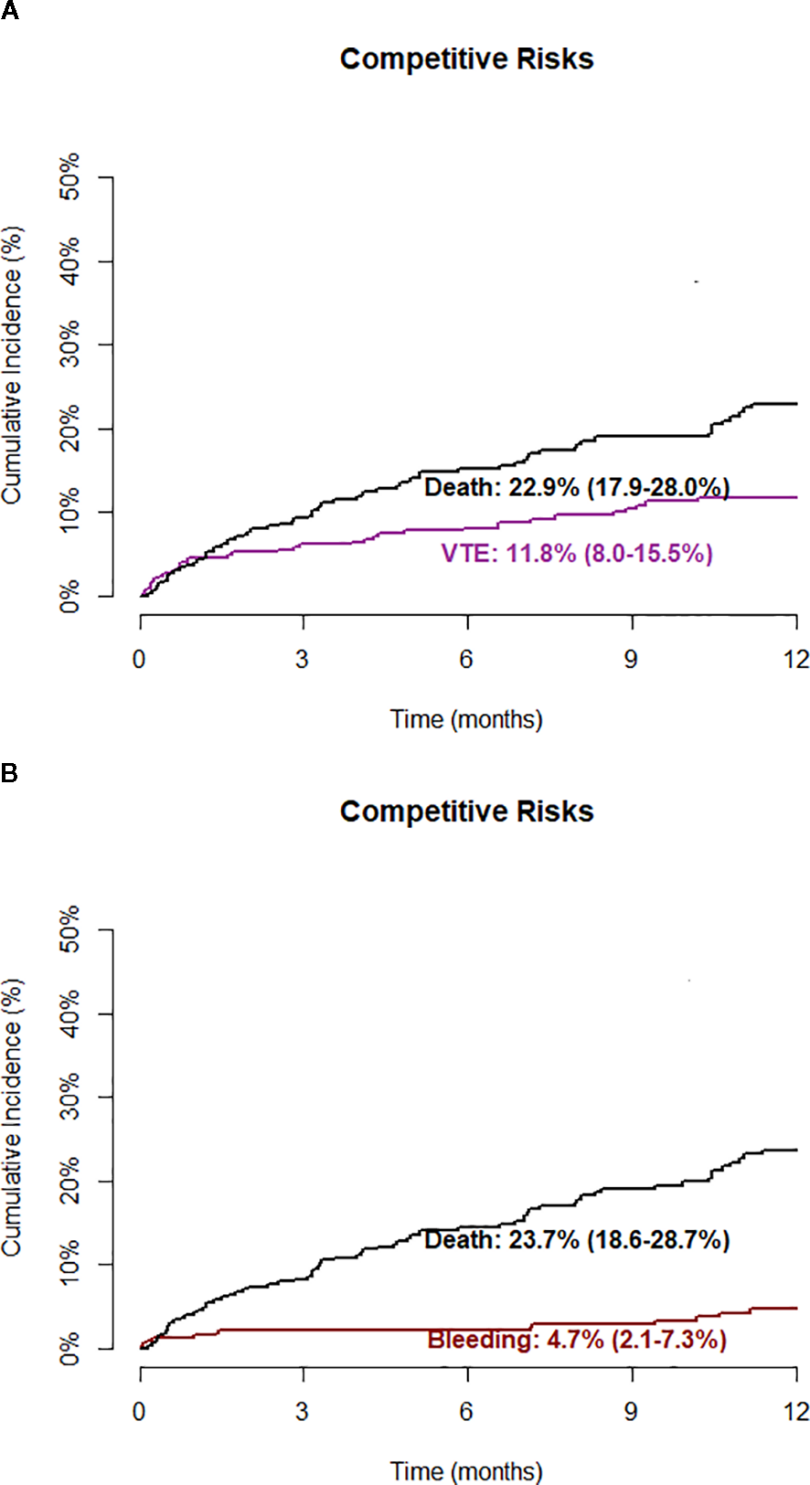
Cumulative incidence for VTE and Bleeding outcomes. **(A)** Comparison between the cumulative incidence of VTE and death. **(B)** Comparison between the cumulative incidence of bleeding and death.

The 12-month cumulative incidence of VTE, accounting for death as a competing risk, was 11.8% (95% CI 8.0 – 15.5%) (**Figure 4A**). The median time from diagnosis to VTE occurrence was 3.9 months (**Figure 1**). Over the same period, the cumulative incidence of major bleeding was 4.7% (95% CI 2.1 – 7.3%) (**Figure 4B**). The median time from diagnosis to bleeding occurrence was 4.3 months (**Figure 2**). The 12-month cumulative incidence of death was 26.2% (95% CI 21.0 – 31.5%). The median follow-up was 10.3 months, and the median time from diagnosis to death was 4.9 months (**Figure 3**).

### 3.2 Risk analysis

In univariate Fine–Gray analysis for VTE, completing high school (vs. ≤ elementary) (SHR 0.32; 95% CI 0.11 – 0.92) and prophylaxis during hospitalization (SHR 0.23; 95% CI 0.10 – 0.79) were associated with reduced risk. Increased risk was observed for prior VTE (SHR 4.45; 95% CI 2.29 – 8.64), prior myocardial infarction or stroke (SHR 3.09; 95% CI 1.36–6.99), immobilization (SHR 2.37; 95% CI 1.21 – 4.67), and corticosteroid use (SHR 3.68; 95% CI 1.12 – 12.14) (**Table 1**). For bleeding, diabetes (SHR 4.11; 95% CI 1.39 – 12.14), chronic kidney failure (SHR 8.90; 95% CI 2.96 – 26.78), heart failure (SHR 6.67; 95% CI 2.18 – 20.38), COPD (SHR 3.91; 95% CI 1.20 – 12.78), hemiparesis/hemiplegia (SHR 5.65; 95% CI 1.55 – 20.56), and immobilization (SHR 7.44; 95% CI 2.30 – 24.03) increased risk (**Table 1**), while radiotherapy was protective (SHR 0.21; 95% CI 0.07 – 0.63) (**Table 2**).

**Table 2:** Univariate SHR regression analyses were conducted on imputed datasets to identify risk factors for VTE and bleeding, and univariate HR analyses were performed for mortality.

Variables	Categories	VTE			Bleeding			Death		
		SHR	95% CI	p	SHR	95% CI	p	HR	95% CI	p
Sex	female (male)	0.93	0.38 - 1.065	0.536	0.88	0.27 - 2.86	0.835	0.80	0.49 - 1.32	0.385
Age group	40 - 60 (< 40)	1.59	0.61 - 4.10	0.340	ND	ND	ND	8.17	2.52 - 26.53	***<0.001***
	> 60 (< 40)	1.76	0.66 - 4.70	0.257	ND	ND	ND	12.05	3.71 - 39.14	***<0.001***
Education level	Elementary school (< Elementary)	0.37	0.10 - 1.33	0.129	1.08	0.18 - 6.54	0.930	0.84	0.44 - 1.60	0.588
	High school (< Elementary)	0.32	0.11 - 0.92	***0.035***	1.15	0.26 - 5.16	0.857	0.24	0.11 - 0.52	***<0.001***
	Higher education (< Elementary)	0.76	0.36 - 1.63	0.481	0.87	0.19 - 3.94	0.859	0.55	0.32 - 0.94	***0.029***
Nutritional status	Underweight (Eutrophic)	1.64	0.52 - 5.19	0.403	1.96	0.18 - 21.58	0.583	0.89	0.39 - 2.02	0.781
	Overweight (Eutrophic)	1.47	0.66 - 3.31	0.346	2.59	0.50 - 13.34	0.255	0.61	0.34 - 1.07	0.086
	Obese (Eutrophic)	0.84	0.32 - 2.22	0.725	3.13	0.62 - 15.89	0.168	0.69	0.39 - 1.23	0.210
Smoking	Yes (No)	1.35	0.65 - 2.80	0.425	0.99	0.27 - 3.63	0.984	1.23	0.74 - 2.05	0.429
Alcoholism	Yes (No)	0.96	0.42 - 2.15	0.912	0.33	0.04 - 2.53	0.286	1.21	0.71 - 2.08	0.485
Hypertension	Yes (No)	1.85	0.95 - 3.59	0.070	1.74	0.59 - 5.17	0.316	1.43	0.91 - 2.25	0.122
Diabetes mellitus	Yes (No)	2.64	1.32 - 5.27	***0.006***	4.11	1.39 - 12.14	***0.011***	0.91	0.49 - 1.68	0.752
Obesity	Yes (No)	0.75	0.34 - 1.67	0.486	2.28	0.77 - 6.73	0.136	1.03	0.62 - 1.70	0.919
Chronic kidney failure	Yes (No)	2.29	0.91 - 5.75	0.078	8.90	2.96 - 26.78	***<0.001***	1.43	0.72 - 2.88	0.310
Congestive heart failure	Yes (No)	2.48	0.98 - 6.24	0.054	6.67	2.18 - 20.38	***0.001***	1.09	0.50 - 2.38	0.824
Previous VTE	Yes (No)	4.45	2.29 - 8.64	***<0.001***	2.94	0.94 - 9.16	0.063	0.95	0.52 - 1.73	0.870
COPD	Yes (No)	2.94	1.31 - 6.59	***0.009***	3.91	1.20 - 12.78	***0.024***	1.22	0.61 - 2.45	0.574
Previous myocardial infarction or stroke	Yes (No)	3.09	1.36 - 6.99	***0.007***	2.69	0.75 - 9.74	0.131	1.26	0.63 - 2.52	0.520
Tumor type	Gliomas NOS (Oligodendroglioma/ Astrocitoma)	0.45	0.10 - 2.11	0.313	0.69	0.10 - 6.55	0.749	12.60	2.76 - 57.51	***0.001***
	GBM (Oligodendroglioma/ Astrocitoma)	1.29	0.59 - 2.83	0.520	1.50	0.40 - 5.63	0.547	21.75	5.32 - 88.91	***<0.001***
	Other (Oligodendroglioma/ Astrocitoma)	1.23	0.34 - 4.57	0.750	1.23	0.13 - 11.36	0.858	ND	ND	ND
Tumor grade	3 or 4 (2)	1.77	0.68 - 4.64	0.245	1.65	0.37 - 7.37	0.513	4.64	1.87 - 11.49	***0.001***
IDH wild-type	Yes (No)	0.52	0.24 - 1.15	0.107	1.14	0.37 - 3.49	0.815	3.33	1.80 - 6.18	***<0.001***
Tumor size	2.5 to 5 cm (< 2.5 cm)	1.02	0.38 - 2.74	0.973	0.87	0.21 - 3.62	0.849	1.29	0.64 - 2.61	0.472
	≥ 5 cm (< 2.5 cm)	1.27	0.50 - 3.25	0.618	0.78	0.19 - 3.22	0.732	1.62	0.83 - 3.19	0.158
Length of hospital days	≥ 7 days (< 7 days)	1.44	0.75 - 2.78	0.278	1.28	0.43 - 3.80	0.656	2.05	1.30 - 3.24	***0.002***
Duration of prophylaxis	3 months (1 month)	0.42	0.10 - 1.78	0.240	ND	ND	ND	ND	ND	ND
	6 months (1 month)	0.35	0.10 - 1.43	0.144	0.21	0.01 - 3.12	0.258	ND	ND	ND
	During hospitalization (1 month)	0.23	0.10 - 0.79	***0.019***	0.43	0.06 - 2.99	0.395	ND	ND	ND
	Others (1 month)	0.26	0.05 - 1.33	0.106	0.66	0.07 - 6.50	0.721	ND	ND	ND
Hemiparesis/hemiplegia	Yes (No)	1.27	0.65 - 2.48	0.482	5.65	1.55 - 20.56	***0.009***	2.07	1.31 - 3.25	***0.002***
Immobilization	Yes (No)	2.37	1.21 - 4.67	***0.012***	7.44	2.30 - 24.03	***0.001***	1.46	0.89 - 2.41	0.133
Corticosteroid use	Yes (No)	3.68	1.12 - 12.14	***0.032***	ND	ND	ND	2.29	1.18 - 4.46	***0.015***
Antiplatelet use	Yes (No)	1.14	0.49 - 2.63	0.763	1.33	0.37 - 4.75	0.662	1.04	0.58 - 1.86	0.901
Radiotherapy	Yes (No)	0.90	0.37 - 2.18	0.823	0.21	0.07 - 0.63	***0.005***	0.23	0.14 - 0.36	***<0.001***
Chemotherapy	Yes (No)	1.44	0.56 - 3.70	0.455	0.36	0.12 - 1.07	0.065	0.29	0.18 - 0.46	***<0.001***
VTE after surgery	Yes (No)	CD	CD	CD	1.14	0.25 - 5.15	0.866	0.82	0.41 - 1.64	0.567
Bleeding after surgery	Yes (No)	NA	NA	NA	CD	CD	CD	3.15	1.44 - 6.87	***0.004***

ND, no data; cell counts were too low to perform statistical analyses; CD, correlated data; NA, not applicable (the outcome occurred prior to the event of interest).Bold and italic p-values indicate statistically significant results.

In univariate Cox analysis (**Table 2**), high school (HR 0.24; 95% CI 0.11 – 0.52) and higher education (HR 0.55; 95% CI 0.32 – 0.94) were associated with lower mortality. Increased risk was observed for gliomas NOS (HR 12.60; 95% CI 2.76 – 57.51), glioblastoma (HR 21.75; 95% CI 5.32 – 88.91), IDH-wildtype (HR 3.33; 95% CI 1.80 – 6.18), hospitalization ≥7 days (HR 2.05; 95% CI 1.30 – 3.24), hemiparesis/hemiplegia (HR 2.07; 95% CI 1.31 – 3.25), corticosteroid use (HR 2.29; 95% CI 1.18 – 4.46), and postoperative bleeding (HR 3.15; 95% CI 1.44 – 6.87). Radiotherapy (HR 0.23; 95% CI 0.14 – 0.36) and chemotherapy (HR 0.29; 95% CI 0.18 – 0.46) were protective (**Table 2**). The original data (i.e., non-imputed values) are presented in **Supplementary Table 6**.

**Figure 5:**
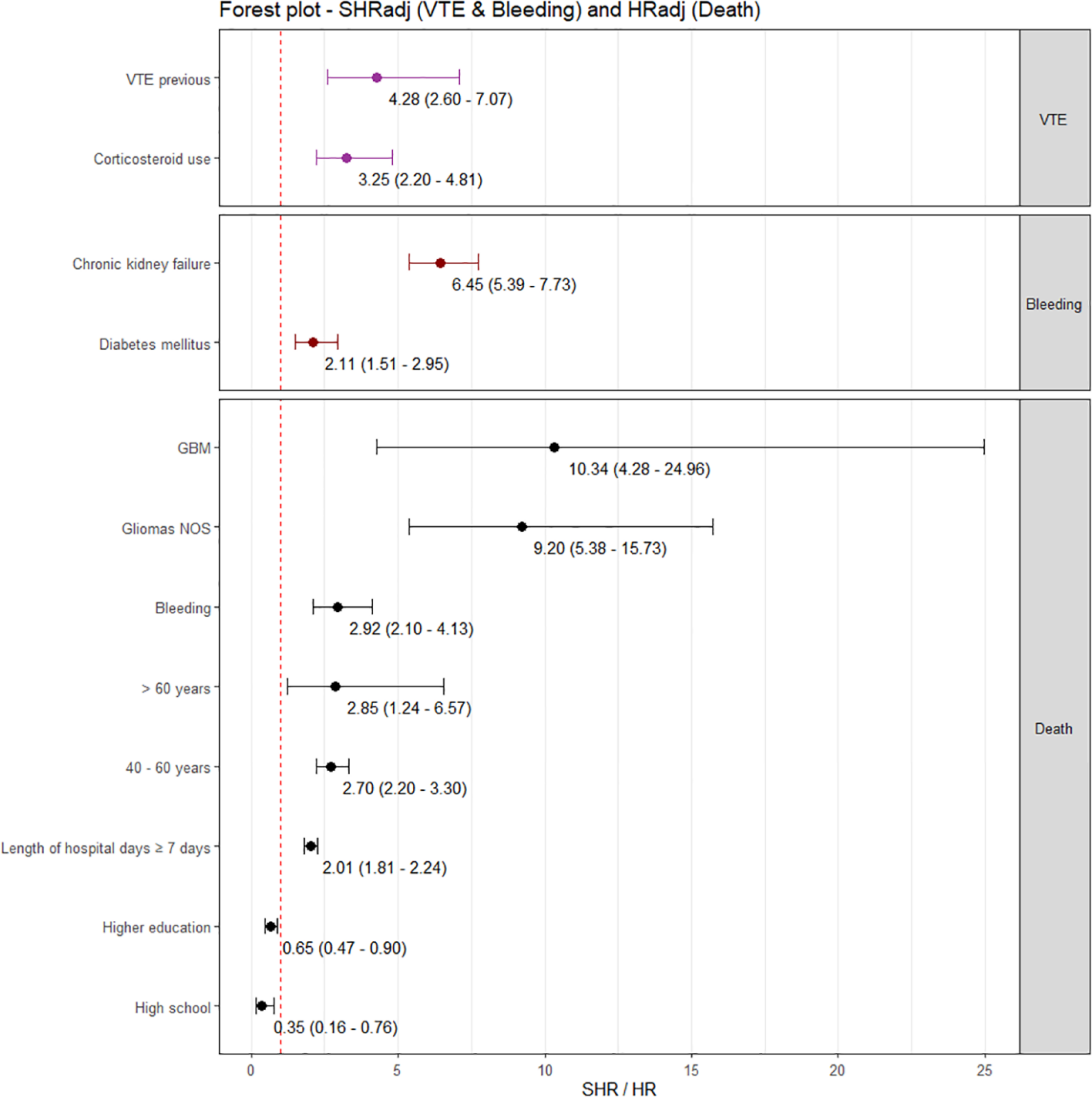
Multiple regression models for the outcomes VTE, Bleeding, and Death. SHR, Subdistribution hazard ratios; HR, Hazard Ratio and respectively 95% CI. The (Venous Thromboembolisms) VTE outcome model was adjusted for tumor type; and the mortality outcome model was adjusted for hypertension, and IDH status.

In the multivariate analysis of independent risk factors for VTE (**Figure 5**), patients with a history of prior VTE had a significantly increased risk, with an adjusted SHRadj of 4.28 (95% CI 2.60 – 7.07). Corticosteroid use was associated with a nearly three-fold increased risk of VTE (SHRadj = 3.25, 95% CI 2.20 – 4.81). The model was adjusted for tumor type. Chronic kidney failure was the main independent predictor of bleeding (SHRadj = 6.45; 95% CI 5.39 – 7.73). Diabetes mellitus was also independently associated with an increased risk of bleeding (SHRadj = 2.11; 95% CI 1.51 – 2.95).

For mortality (**Figure 5**), age was an independent predictor, with a biological gradient. Patients aged 40–60 years and ≥60 years had higher risks compared with those <40 years (HRadj = 2.70; 95% CI 2.20–3.30 and HRadj = 4.13; 95% CI 2.14 – 6.57, respectively). Gliomas NOS and GBM were also associated with markedly increased mortality (HRadj = 9.20; 95% CI 2.34 – 15.73 and HRadj = 10.73; 95% CI 2.44 – 24.96, respectively). Length of hospital stay ≥7 days was associated with higher mortality (HRadj = 2.01; 95% CI 1.28 – 3.23), as was post-surgical bleeding (HRadj = 2.44; 95% CI 1.10 – 5.46). Educational level was independently associated with mortality, with higher education (HRadj = 0.65; 95% CI 0.47 – 0.90) and high school education (HRadj = 0.35; 95% CI 0.16 – 0.76) showing a protective effect compared with lower educational levels. Models were adjusted for hypertension, and IDH status.

Further stratified analyses were conducted in two ways: first, among all patients with grade 3 and 4 gliomas, including both IDH-mutant tumors and GBM (GBM; IDH-wildtype grade 4), and second, in a subgroup analysis restricted to patients with GBM. This approach allowed us to account for prognostic heterogeneity while also providing a more detailed assessment of mortality risk within the clinically distinct GBM subgroup, without relying solely on tumor subtype as a predictor in a global model.

For GBM, at 12 months, the cumulative incidence of death varied across subgroups, being lowest among patients with high school education (21.58%; 95% CI: 5.62–34.84) and higher in other educational groups. Mortality was also higher in patients with Length of hospital stay ≥7 days (47.45% vs. 40.60%) and in those with postoperative bleeding (74.60% vs. 41.96%). In this subgroup, the multivariable Cox model with robust variance estimates showed that secondary education was associated with a significant reduction in the hazard of death (HRadj = 0.33; 95% CI 0.17 – 0.65). Length of hospital stay ≥7 days remained independently associated with increased mortality risk (HRadj = 1.46; 95% CI 1.41 – 1.52), as did the occurrence of bleeding, which more than doubled the hazard of death (HRadj = 2.60; 95% CI 1.98 – 3.42). Univariate analyses are detailed in **Supplementary Table 7**. Notably, multivariable modeling was performed only among GBM tumors due to the number of events.

At 12 months, the cumulative incidence of death among patients with grade 3 and 4 gliomas, including both IDH-mutant tumors and GBM varied across subgroups. Mortality increased with age, from 7.28% (<40 years) to 35.29% (40–60 years) and 40.65% (>60 years). Lower educational level was associated with higher mortality (45.25%), whereas patients with high school education had lower estimates (13.13%). By tumor type, GBM showed the highest mortality (43.75%), followed by gliomas NOS (22.45%) and oligodendroglioma/astrocytoma (4.17%). IDH wild-type tumors had higher mortality compared to IDH-mutant tumors (37.14% vs. 17.99%). Length of hospital stay ≥7 days was associated with increased mortality (38.10% vs. 27.41%), and postoperative bleeding showed the highest estimates (63.33% vs. 30.65%), although with wide confidence intervals (**Table 3**).

**Table 3:** Cumulative incidence analysis and multivariable Cox regression model for patients diagnose0d with GBM tumors and tumor grade 3 or 4.

Variables	Categories	Cumulative incidence (12-months)		HRadj	95% CI	p
		%	95% CI			
GBM						
	< Elementary	54.01	35.41 - 67.25	1.0		
Education level	Elementary school	47.55	20.37 - 65.46	1.05	0.90 - 1.22	0.536
	High school	21.58	5.62 - 34.84	0.33	0.17 - 0.65	***0.001***
	Higher education	48.16	30.96 - 61.08	0.73	0.40 - 1.34	0.312
Length of hospital days	< 7 days	40.60	28.19 - 50.86	1.0		
	≥ 7 days	47.45	33.16 - 58.68	1.46	1.41 - 1.52	***<0.001***
Bleeding after surgery	No	41.96	32.64 - 49.99	1.0		
	Yes	74.60	16.20 - 92.30	2.60	1.98 - 3.42	***<0.001***
Tumor grade 3 or 4						
Age group	< 40	7.28	0.00 - 14.90	1.0		
	40 a 60	35.29	24.35 - 44.65	1.80	1.51 - 2.15	***<0.001***
	> 60	40.65	28.49 - 50.74	1.88	0.63 - 5.64	0.257
	< Elementary	45.25	30.31 - 56.99	1.0		
Education level	Elementary school	37.69	17.90 - 52.72	1.05	0.80 - 1.37	0.738
	High school	13.13	3.32 - 21.95	0.28	0.15 - 0.53	***<0.001***
	Higher education	33.31	21.10 - 43.63	0.69	0.54 - 0.88	***0.003***
	Oligodendroglioma/ Astrocitoma	4.17	0.00 - 11.84	1.0		
Tumor type	Gliomas NOS	22.45	4.86 - 36.79	8.49	1.80 - 39.97	***0.007***
	GBM	43.75	34.64 - 51.60	11.40	3.11 - 41.81	***<0.001***
IDH wild-type	Yes	37.14	28.94 - 44.40	1.0		
	No	17.99	6.78 - 27.85	1.35	1.19 - 1.54	***<0.001***
Length of hospital days	< 7 days	27.41	18.89 - 35.04	1.0		
	≥ 7 days	38.10	26.81 - 47.64	1.76	1.56 - 1.98	***<0.001***
Bleeding after surgery	No	30.65	23.86 - 36.82	1.0		
	Yes	63.33	4.05 - 85.99	2.44	1.73 - 3.44	***<0.001***

Bold and italic p-values indicate statistically significant results.

In the multivariable Cox model among patients with grade 3 and 4 gliomas, including both IDH-mutant tumors and GBM, higher educational level was independently associated with lower mortality (high school: HRadj = 0.28; 95% CI 0.15 – 0.53; higher education: HRadj = 0.69; 95% CI 0.54 – 0.88). Patients aged 40–60 years had increased risk of death (HRadj = 1.80; 95% CI 1.51 – 2.15). Tumor subtype was associated with higher hazards observed for gliomas NOS (HRadj = 8.49; 95% CI 1.80 – 39.97) and GBM (HRadj = 11.40; 95% CI 3.11 – 41.81). IDH wild-type status was associated with increased mortality (HR = 1.35; 95% CI 1.19 – 1.54). Length of hospital stay ≥7 days (HRadj = 1.76; 95% CI 1.56 – 1.98) and postoperative bleeding (HRadj = 2.44; 95% CI 1.73 – 3.44) were independently associated with increased mortality (**Table 3**). Univariate analyses are detailed in **Supplementary Table 8**.

## 4 Discussion

This multicenter study presents evidence regarding risk factors for VTE, bleeding, and mortality among patients with CNS tumors. Data were collected from nine hospitals across distinct Brazilian regions, representing both oncology-focused and general care settings. The inclusion of an ethnically diverse population, within a country marked by substantial geographic and demographic heterogeneity, enhances the relevance and applicability of the epidemiological and clinical insights generated. The 12-month cumulative incidence of VTE was 11.8%, consistent with previous reports in patients with CNS tumors (23). Our cohort was predominantly composed of men and individuals older than 40 years, demographics that have been repeatedly associated with increased VTE risk in this population. In addition, metabolic and cardiovascular comorbidities, including overweight/obesity, hypertension, diabetes, and smoking, were highly prevalent, further supporting their established contribution to prothrombotic risk (3, 8, 13).

The median time to postoperative events was less than four months, reinforcing that the early postoperative period represents a window of heightened thrombotic vulnerability, as similarly described in prior studies (2, 7, 8). The clinical burden of VTE in gliomas is substantial. After craniotomy for tumor resection, postoperative DVT occurs in 3.9%, while silent PE is identified in up to 26% of patients (1, 3, 17, 24). VTE rates increase with glioma grade from 5.2% in grade II to 6.3% in grade III and 6.8% in grade IV. In adult-type diffuse gliomas, cumulative VTE incidence reaches 12%, 18%, and 20% at 6, 12, and 24 months, respectively (7, 8, 10, 12, 25, 26). In GBM, VTE is linked to a 30% increase in two-year mortality, poorer neurological outcomes (lower modified Rankin and Glasgow Outcome Scale scores), and extended hospital stay (median 22 versus 5 days without DVT) (1, 3, 25).

At the molecular level, most patients who developed VTE harbored IDH wild-type tumors (73% in our cohort). While this finding is consistent with prior reports showing that patients with IDH wild-type gliomas or GBM may exhibit up to a threefold increased risk of DVT compared to those with IDH-mutant tumors (5), it should be interpreted with caution. IDH wild-type tumors largely overlap with higher-grade gliomas, which are more frequently diagnosed in older patients, both well-established risk factors for VTE. In our study, among patients with grade 3 and 4 tumors, approximately 40% were older than 60 years, whereas only 18% were younger than 40 years, reinforcing the potential contribution of age and tumor aggressiveness to the observed association. Therefore, the increased VTE risk may reflect, at least in part, the underlying biological severity and clinical profile of these tumors rather than an isolated effect of IDH status. Nevertheless, a direct mechanistic link remains plausible, as IDH mutations have been shown to suppress tissue factor expression, a key initiator of the coagulation cascade, thereby attenuating thrombotic potential (5, 8, 12, 13).

In addition to tumor-specific mutations, the broader molecular risk is underscored by a high heritability of VTE, estimated at up to 60%. This genetic predisposition is frequently linked to gain-of-function mutations, such as Factor V Leiden (FVL) and the prothrombin G20210A mutation (PGM), as well as deficiencies in natural anticoagulants like antithrombin, protein C, and protein S. Notably, while these markers are central to the thrombophilic profile, MTHFR polymorphisms are not associated with VTE risk and are excluded from recommended molecular panels (27).

Only a prior history of VTE and corticosteroid use emerged as independent predictors of VTE. Although only 4.8% of patients had a previous event, nearly 30% experienced recurrence, reinforcing the well-established link between malignancy and recurrent thrombosis. Recurrence rates in malignancy vary widely according to tumor-driven biological factors, with evidence implicating leukocyte and platelet counts, P-selectin, prothrombin fragment 1 + 2, factor VIII activity, and D-dimer in modulating thrombotic risk (1, 28). Kyrle et al. (29) reported a one-year recurrence rate of 5.2% (95% CI: 3.2–7.2), largely driven by persistent prothrombotic conditions, including active malignancy, thrombophilia, and endothelial dysfunction. Consistent with this, the 2023 ASH guidelines suggest that patients with cancer at low or intermediate risk who have a family history of VTE, testing for a panel of hereditary thrombophilias may be considered to further individualize management (27). Elevated D-dimer after anticoagulation discontinuation is a strong predictor of recurrence, supporting consideration of prolonged anticoagulation in selected high-risk individuals (1, 29).

Corticosteroid therapy conferred an approximately threefold increased VTE risk, corroborating previous findings (13, 30, 31). The underlying mechanisms are likely multifactorial. According to Orešković et al. (31), corticosteroids, particularly dexamethasone, commonly used in GBM patients to manage peritumoral edema, may induce a hypercoagulable state via steroid-induced hyperglycemia and activation of the intrinsic pathway, evidenced by the inverse correlation between glycated hemoglobin (HbA1c) and activated partial thromboplastin time (aPTT). Moreover, in neurosurgical populations, pre- and perioperative corticosteroid use has been linked to impaired clot contractility, a marker of imminent VTE risk (30). The persistence of corticosteroids as an independent predictor after adjusting for tumor type indicates a prothrombotic effect that extends beyond their use in high-grade tumor management.

Other relevant findings of our study included a high proportion of VTE observed among patients with GBM (63.4%) and high-grade tumors in general (87.8%), highlighting the strong influence of tumor aggressiveness on thrombotic predisposition. Consistent with prior research, GBM is associated with VTE rates up to 30% across the disease course and represents a leading cause of morbidity and mortality (3, 5, 13, 26, 32). These data reinforce the biological thrombotic phenotype of high-grade gliomas. Although high-grade gliomas and GBM accounted for most VTE cases, these variables did not reach statistical significance in our model, despite an almost threefold increased risk. This pattern aligns with our molecular findings, in which IDH wild-type tumors, frequently overlapping with higher-grade gliomas and older age groups, accounted for most VTE events, suggesting that the observed association may be largely driven by tumor aggressiveness and patient age rather than an isolated effect of IDH status.

For bleeding, our study observed a 12-month cumulative incidence of 4.7%, which is consistent with previous studies reporting bleeding rates between 2% and 10% in patients with CNS tumors (25, 33, 34). Notably, we found that 7.3% of patients experienced a bleeding event after VTE. Moreover, half of the events occurred within the first four months. Although this percentage is relatively low, long-term thromboprophylaxis is not routinely administered to prevent VTE events. A high-risk profile was observed among patients who experienced bleeding, predominantly those with high-grade tumors, particularly GBM, with an IDH-wild-type genotype, aligning with evidence that these subtypes carry the greatest hemorrhagic susceptibility (35, 36). None of the affected patients had a history of thrombosis or prior bleeding, suggesting an intrinsic tumor- and treatment-related risk. Thus, anticoagulation has been associated with increased bleeding risk in malignant brain tumors, representing a major barrier to routine extended thromboprophylaxis in this setting (17).

The predominance of intracranial bleeding highlights the concern regarding ICH, making pharmacological prophylaxis controversial after surgery (12, 30). ICH remains the key restraint on anticoagulation use after neurosurgery. As a result, clinicians often rely on mechanical prophylaxis or limit pharmacological prophylaxis to hospitalization, as seen in our cohort since half of the bleeding cases received only mechanical prophylaxis, and most had prophylaxis limited to hospital stay, increasing post-discharge VTE risk. However, this conservative approach leaves patients at elevated risk of VTE following discharge.

Nevertheless, early initiation of LMWH prophylaxis, such as enoxaparin within 72 hours after surgery, has demonstrated safety and efficacy in preventing lower-limb DVT without increasing rates of hematoma, ischemic stroke, PE, or mortality (1, 3, 37–40). DOACs, particularly apixaban and rivaroxaban, have emerged as promising alternatives to LMWH, with comparable efficacy in VTE prevention and potentially lower rates of major bleeding and ICH in patients with CNS tumors. Current guidelines from the American Society of Hematology (ASH) and the International Initiative on Thrombosis and Cancer (ITAC) endorse LMWH and DOACs for the treatment and prevention of cancer-associated thrombosis (CAT) in selected patients with primary and metastatic brain tumors (26, 33, 34, 41).

Chronic kidney disease (CKD) emerged as the sole independent risk factor for bleeding. CKD is common among cancer patients and is associated with poorer outcomes (42, 43). Renal impairment alters the pharmacokinetics of several drugs, including chemotherapeutic agents and anticoagulants such as low-molecular-weight heparin (LMWH), which is predominantly renally excreted (42–44). CKD has been linked to a progressive increase in major bleeding, particularly in patients treated with LMWH and with an estimated glomerular filtration rate (eGFR) <30 mL/min. This interaction may explain why CKD was an independent predictor of bleeding in our cohort, outweighing other established risk factors. Additionally, CKD-related chronic inflammation, oxidative stress, and endothelial dysfunction, which in themselves may contribute to both thrombotic and hemorrhagic risk (45, 46).

A well-established association exists between diabetes and CKD, which could suggest a confounding effect. However, our multivariable analysis demonstrated that both conditions remained independently associated with bleeding, reinforcing the importance of careful adjustment for confounders when assessing risk factors. Diabetes is a major contributor to CKD, markedly increasing the risk of diabetic nephropathy (45, 47, 48), while CKD itself is a recognized determinant of bleeding risk, particularly in patients with cancer and VTE (44). This elevated risk is largely driven by uremia-related hemostatic dysfunction and reduced clearance of renally excreted anticoagulants, such as low-molecular-weight heparin. Supporting this, Hasegawa et al. reported that both diabetes and CKD were independent predictors of major bleeding after percutaneous renal biopsy, suggesting distinct underlying mechanisms. Similarly, other studies have demonstrated an additive effect between these conditions, with diabetic kidney disease associated with increased mortality and an even greater risk when both conditions coexist (49).

However, in patients with central nervous system (CNS) tumors, the relationship between diabetes, CKD, and bleeding remains less clear. Although CKD is a common complication of diabetes, a prior study (41) in patients with glioblastoma did not identify CKD as a mediator of increased major bleeding risk, suggesting that these interactions may vary according to tumor type and patient profile. In contrast, Orešković et al. (31) proposed that hyperglycemia, particularly in highly proliferative glioblastoma, may disrupt coagulation homeostasis through activation of the intrinsic pathway. Additionally, cancer and CKD share a bidirectional relationship: CKD promotes a pro-inflammatory and oxidative stress milieu that may favor oncogenesis, while nephrotoxic anticancer therapies can induce or worsen renal dysfunction (42, 43, 45). Importantly, patients with CKD are frequently excluded from clinical trials, perpetuating a significant evidence gap in one of the most vulnerable subgroups in neuro-oncology (43).

No significant association was found between VTE and mortality, in line with previous studies (8, 50). However, other authors have reported that VTE-related mortality may be the second leading cause of death in this population, after tumor progression (3, 5, 13). In our study, about 30% of patients with postoperative VTE had a prior VTE, which may have led to more cautious prophylaxis and contributed to the absence of a causal link. Postoperative bleeding independently increased mortality risk nearly threefold, which is consistent with previous evidence identifying this complication as a major determinant of adverse outcomes (16, 34).

Tumor severity, particularly in gliomas NOS and GBM, was associated with increased mortality. GBMs are highly infiltrative tumors often located in eloquent brain regions, limiting surgical resection. Their genetic and epigenetic heterogeneity, along with an immunosuppressive microenvironment, contributes to therapeutic resistance and poor outcomes. Key alterations such as EGFR amplification, PTEN deletion, and TERT promoter mutations are linked to aggressive behavior (51). Favorable prognostic factors include low Ki-67, MGMT promoter methylation, IDH mutations, and absence of p53 overexpression, while longer symptom duration may reflect slower tumor progression and improved survival (52).

This impact is modulated by tumor histology, extent of resection, and baseline clinical status. Among patients with bleeding, nearly 90% had grade 3 and 4 GBM, predominantly IDH–wildtype, with high rates of hypertension and diabetes. Bleeding may act as an intermediate factor, as intracranial hemorrhage during anticoagulation in GBM is associated with poor prognosis, highlighting the narrow balance between preventing VTE and avoiding ICH (17, 25, 41, 53). Histopathology remains a key determinant of outcomes, supporting the need for early diagnosis and individualized management in high-grade gliomas. Stratified analyses by tumor subtype were not feasible due to the limited number of events in most subgroups, which may have affected the ability to detect subtype-specific associations. Referral bias should also be considered, as this tertiary center-based cohort may overrepresent more severe cases, limiting generalizability.

Age was an independent predictor of mortality, demonstrating a biological gradient, with patients aged 40–60 years and >60 years experiencing significantly worse outcomes. This aligns with the biological gradient theory, whereby advancing age is associated with diminishing resilience and survival (26). Increased mortality in older adults reflects greater comorbidity burden, reduced physiological reserve, and limited tolerance to surgical, infectious, or oncologic stressors. Conversely, younger patients generally exhibit better functional status and are more frequently eligible for prolonged and aggressive treatment protocols, contributing to improved survival (8, 54).

Hospitalization ≥7 days was independently associated with mortality, supporting its role as a proxy for disease severity and care complexity. Although length of stay has a bidirectional relationship with clinical deterioration, the presence of neurological deficits, immobility and comorbid conditions may amplify the risk of VTE and bleeding, contributing to poorer outcomes (3, 55, 56). Thus, extended hospitalization may serve as an early warning indicator to identify high-risk patients who require targeted preventive and supportive interventions. Given the mortality differences between oncology-specialized and general hospitals, this variable was included in the multivariable model to adjust for potential confounding effects. This variation may be attributable to a higher proportion of patients with advanced malignancy, more severe comorbidities, or the need for complex therapeutic procedures in specialized centers. The model accounted for tumor type, noting that all GBM cases were classified as grade 3 or 4, IDH-wildtype, with hypertension being the most prevalent comorbidity.

Educational level appears to play a relevant role in mortality, even in severe conditions such as gliomas, highlighting the real-world impact of socioeconomic disparities in Brazil. These findings are consistent with prior evidence suggesting that factors such as insurance coverage are associated with improved prognosis (54). This relationship may be explained by differences in socioeconomic resources, as patients with higher educational attainment are more likely to benefit from stronger family support and more timely and consistent access to healthcare services, including specialized centers and appropriate treatments, which may facilitate earlier diagnosis and improved outcomes.

Given the well-established prognostic heterogeneity across glioma subtypes, we performed stratified analyses restricted to high-grade tumors, as well as a separate analysis for GBM, to evaluate the role of clinical and socioeconomic factors on mortality within more homogeneous settings. In our cohort, GBM accounted for the highest burden of adverse events and maintained the aggressive behavior widely described in the literature, with limited overall survival and high 12-month mortality, as previously reported (3, 5, 13, 51). This approach reduced the isolated impact of tumor biology and allowed the exploration of additional prognostic determinants.

Among patients with high-grade tumors, the 12-month cumulative incidence of death varied significantly across strata. Lower mortality was observed among individuals with higher educational level, whereas increased risk was seen in those with prolonged hospitalization and postoperative bleeding. This pattern was consistent with the overall analysis, reinforcing the robustness of these associations regardless of tumor grade. These findings are in line with prior studies showing that hospital-related factors, perioperative complications, and social determinants significantly influence outcomes in neuro-oncology (8, 26, 54).

Educational level remained independently associated with a reduced risk of death, supporting evidence that socioeconomic determinants influence access to specialized care, treatment adherence, and clinical outcomes (54). In contrast, hospitalization ≥7 days and postoperative bleeding emerged as strong predictors of mortality, with bleeding showing the largest effect size. These results are consistent with previous studies identifying intracranial hemorrhagic complications as major determinants of poor prognosis in patients with brain tumors (16, 34).

Despite the central role of tumor biology, particularly in IDH wild-type tumors with aggressive histopathological features, our findings indicate that clinical course variables also play a significant role in mortality risk (13). The management of perioperative complications, especially intracranial bleeding, has been consistently associated with worse clinical outcomes and increased mortality (17, 25, 41). Additionally, length of hospital stay, often interpreted as a proxy for disease severity and care complexity, has also been linked to adverse outcomes (3, 55, 56).

Overall, these results reinforce that mortality in high-grade gliomas, including IDH-mutant grade 3 and 4 tumors and GBM (IDH-wildtype grade 4), arises from a multifactorial interplay between biological aggressiveness, clinical course, and social determinants. While molecular and histopathological features remain central to prognostic stratification, potentially modifiable factors, such as complication prevention and optimization of hospital care, as well as socioeconomic disparities, play a relevant role and should be considered in management strategies and health policies.

This study has limitations, primarily related to missing data. Key biochemical variables, such as inflammatory and coagulation markers (e.g., D-dimer and platelet counts), were unavailable, as they are not routinely collected in many healthcare settings. Their absence may have resulted in residual confounding, limited multivariable adjustment, and reduced exploration of underlying biological mechanisms. Additionally, the lack of molecular characterization (e.g., MGMT promoter methylation, TERT mutations, and EGFR alterations) restricted more precise tumor stratification and may have affected prognostic interpretation across biologically distinct subtypes.

Missing data in several clinical variables required multiple imputation (MICE), assuming data were missing at random. The imputation model incorporated variables related to both outcomes and missingness, with no changes observed in the direction of effect estimates. Nonetheless, the proportion of imputed data ranged from 3.0% to 25.7% (**Supplementary Tables 4 and 5**). These limitations also reflect structural constraints, including limited resources that impact the routine collection of detailed clinical and molecular data in real-world settings. Therefore, findings should be interpreted with caution, and future studies incorporating comprehensive biomarker data are warranted.

The retrospective design introduces potential survival bias; however, the short median time to death (5 months) highlights disease severity. Follow-up was limited to one year, and 12.6% of patients were lost after six months, reflecting operational challenges in longitudinal studies. Finally, disparities in access to specialized CNS tumor care and referral patterns to tertiary centers may introduce selection bias and limit generalizability (7). To mitigate inter-institutional variability, cluster-robust standard errors were applied, improving model stability.

A strength of this study was the standardized data collection protocol implemented across all participating sites. A Manual of Procedures (MOP) was developed to guide medical record abstraction, REDCap data entry, and participant follow-up. Online training ensured protocol compliance, and all staff were required to hold Good Clinical Practice (GCP) certification. For statistical analysis, a competing risk model was applied to generate adjusted risk estimates, minimizing the overestimation bias commonly observed in traditional survival analyses. By accounting for alternative outcomes (e.g., death), this approach yielded more accurate and clinically relevant estimates of cumulative incidence.

## 5 Conclusion

In this Brazilian multicenter cohort, VTE showed a relevant and early incidence following surgery for gliomas and was mainly driven by clinical factors (prior VTE, corticosteroid use) and tumor aggressiveness. Bleeding, although less frequent, had a significant prognostic impact, with chronic kidney disease emerging as the main determinant of bleeding risk, and the presence of diabetes mellitus. The lack of an independent association between VTE and mortality, contrasted with the strong effects of bleeding and tumor severity, as well as demographic factors such as age and educational level, highlights the narrow therapeutic window of anticoagulation in neuro-oncology. These data support a personalized approach to thromboprophylaxis that integrates tumor characteristics, comorbidities, and renal function, and emphasize the need to improve access to specialized care and risk stratification in resource-limited settings.

## Data Availability

The raw data supporting the conclusions of this article will be made available by the authors, without undue reservation.
