# Supplementary material for: Venous thromboembolism and secondary outcomes of bleeding and mortality in patients with gliomas: a multicenter cohort study
Source: Front Oncol. 2026 May 21;16:1771694. doi: 10.3389/fonc.2026.1771694 (PMC13233262; doi:10.3389/fonc.2026.1771694)
Supplement: Supplementary file 1 [file Table1.docx]

Supplementary Table 1 – Patient with diagnosis between 2021 and 2023 across nine Brazilian centers.

| **Institution** | **City/State** |
| --- | --- |
| BP – A Beneficência Portuguesa de São Paulo - The Coordinating Center in São Paulo city | São Paulo/São Paulo |
| Hcor – Associação Beneficente Síria | São Paulo/São Paulo |
| ICESP - Instituto do Câncer do Estado de São Paulo | São Paulo/São Paulo |
| AC Camargo Cancer center | São Paulo/São Paulo |
| Hospital de Amor | Barretos/São Paulo |
| Santa Casa de Santa Izabel | Salvador/Bahia |
| Liga Norte Contra o Câncer | Natal/Rio Grande do Norte |
| INCA - Instituto Nacional do Câncer | Rio de Janeiro/Rio de Janeiro |
| PUC - Hospital da Pontifícia Universidade Católica | Porto Alegre/Rio Grande do Sul |
