# Supplementary material for: Venous thromboembolism and secondary outcomes of bleeding and mortality in patients with gliomas: a multicenter cohort study
Source: Front Oncol. 2026 May 21;16:1771694. doi: 10.3389/fonc.2026.1771694 (PMC13233262; doi:10.3389/fonc.2026.1771694)
Supplement: Supplementary file 2 [file Table2.docx]

Supplementary Table 2 - Other tumors (n= 26).

| **Tumor classification** | **n** |
| --- | --- |
| ***Ependymal tumors*** | ***12*** |
| Ependymoma | 11 |
| Ependymoma anaplastic | 1 |
| ***Embryonal tumors*** | ***5*** |
| Medulloblastoma | 5 |
| ***Circumscribed astrocytic gliomas*** | ***2*** |
| Xantoastrocitoma pleomorphic | 2 |
| ***Mesenchymal non-meningothelial tumor*** | ***2*** |
| Solitary fibrous tumor | 2 |
| ***Meningioma (Grau III)*** | ***4*** |
| ***Pineal tumors*** | ***1*** |
| Neuroepithelial neoplasm | 1 |
