# Supplementary material for: Venous thromboembolism and secondary outcomes of bleeding and mortality in patients with gliomas: a multicenter cohort study
Source: Front Oncol. 2026 May 21;16:1771694. doi: 10.3389/fonc.2026.1771694 (PMC13233262; doi:10.3389/fonc.2026.1771694)
Supplement: Supplementary file 3 [file Table3.docx]

Supplementary Table 3 – Number and percentage of patients by type of chemotherapeutic or immunotherapeutic agent. TROMBOGLIO 2021-2023.

| **Chemotherapy (n=256*)** | **n** | **%** |
| --- | --- | --- |
| Temozolomida | 236 | 92.2 |
| Lomustina | 26 | 10.2 |
| Bevacizumab | 15 | 5.9 |
| Procarbarzina | 16 | 6.3 |
| Vincristina | 15 | 5.9 |
| Cisplatina | 5 | 2.0 |
| Irinotecano | 4 | 1.6 |
| Etoposídeo | 3 | 1.2 |
| Carmustina | 1 | 0.4 |
| Ciclofosfamida | 2 | 0.8 |
| Carboplatina | 1 | 0.4 |
| Doxiciclina | 1 | 0.4 |
| Mebendezol | 1 | 0.4 |
| Osimertinib | 1 | 0.4 |

* Eight missing information
