# Supplementary material for: Venous thromboembolism and secondary outcomes of bleeding and mortality in patients with gliomas: a multicenter cohort study
Source: Front Oncol. 2026 May 21;16:1771694. doi: 10.3389/fonc.2026.1771694 (PMC13233262; doi:10.3389/fonc.2026.1771694)
Supplement: Supplementary file 4 [file Table4.docx]

Supplementary Table 5 – Number and percentage of participants with CNS according to tumor, and clinical characteristics.

| Variables | Categories | Total (334) | VTE (n=41) | Bleeding (n=14) | Death (n=96) |
| --- | --- | --- | --- | --- | --- |
|  |  | n (%) | n (%) | n (%) | n (%) |
| Tumor type | Oligodendroglioma/ Astrocitoma | 94 (28.1) | 10 (24.4) | 3 (21.4) | 4 (4.2) |
|  | Gliomas NOS* | 44 (13.2) | 2 (4.9) | 1 (7.1) | 12 (12.5) |
|  | GBM | 170 (50.9) | 26 (63.4) | 9 (64.4) | 79 (82.3) |
|  | Other | 26 (7.8) | 3 (7.3) | 1 (7.1) | 1 (1.0) |
| Tumor grade | 2 | 75 (22.5) | 5 (12.2) | 2 (14.3) | 7 (7.3) |
|  | 3 or 4 | 259 (77.5) | 36 (87.8) | 12 (85.7) | 89 (92.7) |
| IDH wild-type | No | 181 (54.2) | 27 (65.9) | 7 (50.0) | 70 (72.9) |
|  | Yes | 107 (32) | 10 (24.3) | 4 (28.8) | 12 (12.5) |
|  | Missing | 46 (13.8) | 4 (9.6) | 3 (21.2) | 14 (14.6) |
| Local tumor | Infratentorial | 16 (4.8) | 2 (4.9) | 0 (0.0) | 3 (3.1) |
|  | Intramedular | 3 (0.9) | 0 (0.0) | 0 (0.0) | 0 (0.0) |
|  | Supratentorial | 314 (94) | 0 (0.0) | 0 (0.0) | 1 (1.0) |
|  | Missing | 1 (0.3) | 39 (95.1) | 14 (100) | 92 (95.9) |
| Tumor size | < 2.5 cm | 43 (12.9) | 6 (14.6) | 3 (21.4) | 5 (5.2) |
|  | 2.5 to 5 cm | 109 (32.6) | 12 (29.3) | 4 (28.6) | 36 (37.5) |
|  | ≥ 5 cm | 130 (38.9) | 17 (41.5) | 5 (35.7) | 40 (41.7) |
|  | Missing | 52 (15.6) | 6 (14.6) | 2 (14.3) | 15 (15.6) |
| Length of hospital days | < 7 days | 140 (41.9) | 14 (34.1) | 5 (35.7) | 36 (37.5) |
|  | ≥ 7 days | 108 (32.3) | 14 (34.1) | 6 (42.9) | 40 (41.7) |
|  | Missing | 86 (25.7) | 13 (31.8) | 3 (21.4) | 20 (20.8) |
| Type of Prophylaxis | None | 20 (6.0) | 1 (2.4) | 0 (0.0) | 5 (5.2) |
|  | Pharmacological | 15 (4.5) | 3 (7.3) | 0 (0.0) | 1 (1.0) |
|  | Mechanical | 85 (25.4) | 9 (22.0) | 7 (50.0) | 27 (28.2) |
|  | Combined | 117 (35) | 10 (24.4) | 4 (28.6) | 39 (40.6) |
|  | Missing | 97 (29.0) | 18 (43.9) | 3 (21.4) | 24 (25.0) |
| Duration of prophylaxis | 1 month | 2 (0.6) | 1 (2.4) | 1 (7.1) | 0 (0.0) |
|  | 3 months | 2 (0.6) | 1 (2.4) | 0 (0.0) | 0 (0.0) |
|  | 6 months | 3 (0.9) | 2 (4.9) | 1 (7.1) | 1 (1.0) |
|  | During hospitalization | 202 (60.5) | 17 (41.5) | 9 (64.4) | 64 (66.7) |
|  | Others | 7 (2.1) | 1 (2.4) | 0 (0.0) | 2 (2.1) |
|  | Missing | 118 (35.3) | 19 (46.4) | 3 (21.4) | 29 (30.2) |
| Presence of hemiparesis/hemiplegia | No | 195 (58.4) | 20 (48.9) | 2 (14.3) | 39 (40.6) |
|  | Yes | 121 (36.2) | 19 (46.3) | 10 (71.4) | 51 (53.1) |
|  | Missing | 18 (5.4) | 2 (4.8) | 2 (14.3) | 6 (6.3) |
| Immobilization | No | 239 (71.6) | 21 (51.2) | 5 (35.7) | 62 (64.6) |
|  | Yes | 40 (12) | 9 (22.0) | 5 (35.7) | 17 (17.7) |
|  | Missing | 55 (16.5) | 11 (26.8) | 4 (28.6) | 17 (17.7) |
| Corticosteroid use | No | 69 (20.7) | 4 (9.8) | 0 (0.0) | 8 (8.3) |
|  | Yes | 249 (74.6) | 36 (87.8) | 14 (100) | 85 (88.6) |
|  | Missing | 16 (4.8) | 1 (2.4) | 0 (0.0) | 3 (3.1) |
| Antiplatelet use | No | 249 (74.6) | 31 (75.6) | 8 (57.2) | 70 (72.9) |
|  | Yes | 18 (5.4) | 4 (9.8) | 1 (7.1) | 3 (3.1) |
|  | Missing | 67 (20.1) | 6 (14.64) | 5 (35.7) | 23 (24.0) |
| Radiotherapy | No | 54 (16.2) | 4 (9.8) | 5 (35.7) | 26 (27.1) |
|  | Yes | 273 (81.7) | 34 (82.9) | 8 (57.2) | 67 (69.8) |
|  | Missing | 7 (2.1) | 3 (7.3) | 1 (7.1) | 3 (3.1) |
| Chemotherapy | No | 70 (21) | 4 (9.8) | 5 (35.7) | 28 (29.2) |
|  | Yes | 257 (76.9) | 34 (82.9) | 8 (57.2) | 65 (67.7) |
|  | Missing | 7 (2.1) | 3 (7.3) | 1 (7.1) | 3 (3.1) |
| Platelet count | n (median) | 317 (230,000) | 39 (182,794) | 13 (180,000) | 94 (207,000) |
|  | Min-Max | 34,000 – 482,000 | 85,000 – 440,000 | 75,000 – 462,000 | 45,000 – 482,000 |
|  | Missing | 17 | 2 | 1 | 2 |
| INR | n (median) | 283 (1.0) | 37 (1.0) | 12 (1.0) | 87 (1.0) |
|  | Min-Max | 0.69 – 11.30 | 0.75 – 10.60 | 0.86 – 1.10 | 0.75 – 10.60 |
|  | Missing | 51 | 4 | 2 | 9 |

* Valid number per category; VTE = Deep vein thrombosis or pulmonary embolism; INR = International Normalized Ratio
