# Supplementary material for: Venous thromboembolism and secondary outcomes of bleeding and mortality in patients with gliomas: a multicenter cohort study
Source: Front Oncol. 2026 May 21;16:1771694. doi: 10.3389/fonc.2026.1771694 (PMC13233262; doi:10.3389/fonc.2026.1771694)
Supplement: Supplementary file 5 [file Table5.docx]

Supplementary Table 6 – Univariate SHR regression analyses were conducted on original datasets to identify risk factors for VTE and bleeding, and univariate HR analyses were performed for mortality.

| Variables | Categories | VTE | | | Bleeding | | | Death | | |
| --- | --- | --- | --- | --- | --- | --- | --- | --- | --- | --- |
|  |  | SHR | 95% CI | p | SHR | 95% CI | p | HR | 95% CI | p |
| Education level | Elementary school (< Elementary) | 0.48 | 0.10 - 2.36 | 0.367 | 1.78 | 0.11 - 29.08 | 0.687 | 0.73 | 0.33 - 1.59 | 0.425 |
|  | High school (< Elementary) | 0.50 | 0.16 - 1.57 | 0.234 | 3.07 | 0.33 - 28.06 | 0.321 | 0.27 | 0.12 - 0.59 | 0.001 |
|  | Higher education (< Elementary) | 1.11 | 0.45 - 2.74 | 0.822 | 2.34 | 0.25 - 21.63 | 0.455 | 0.45 | 0.24 - 0.84 | 0.012 |
| Nutritional status | Underweight (Eutrophic) | 1.66 | 0.52 - 5.26 | 0.389 | 1.94 | 0.18 - 21.83 | 0.575 | 0.90 | 0.40 - 2.05 | 0.806 |
|  | Overweight (Eutrophic) | 1.45 | 0.65 - 3.26 | 0.366 | 2.56 | 0.50 - 13.16 | 0.261 | 0.60 | 0.34 - 1.06 | 0.077 |
|  | Obese (Eutrophic) | 0.84 | 0.32 - 2.24 | 0.734 | 3.16 | 0.62 - 16.00 | 0.165 | 0.70 | 0.39 - 1.24 | 0.220 |
| Smoking | Yes (No) | 1.35 | 0.60 - 3.02 | 0.463 | 1.22 | 0.33 - 4.54 | 0.766 | 1.42 | 0.83 - 2.43 | 0.207 |
| Alcoholism | Yes (No) | 0.81 | 0.31 - 2.10 | 0.659 | ND | ND | ND | 1.41 | 0.80 - 2.47 | 0.238 |
| Hypertension | Yes (No) | 1.78 | 0.91 - 3.49 | 0.092 | 1.79 | 0.60 - 5.31 | 0.294 | 1.42 | 0.90 - 2.25 | 0.132 |
| Diabetes mellitus | Yes (No) | 3.12 | 1.55 - 6.26 | 0.001 | 4.58 | 1.55 - 13.54 | 0.006 | 0.75 | 0.37 - 1.51 | 0.424 |
| Obesity | Yes (No) | 0.79 | 0.34 - 1.84 | 0.588 | 2.20 | 0.70 - 6.88 | 0.174 | 1.12 | 0.66 - 1.88 | 0.683 |
| Chronic kidney failure | Yes (No) | ND | ND | ND | 54.95 | 9.56 - 315.96 | <0.001 | ND | ND | ND |
| Congestive heart failure | Yes (No) | ND | ND | ND | 18.90 | 2.91 - 122.79 | 0.002 | ND | ND | ND |
| Previous VTE | Yes (No) | 13.58 | 6.67 - 27.64 | <0.001 | ND | ND | ND | 0.75 | 0.23 - 2.38 | 0.622 |
| COPD | Yes (No) | 3.15 | 0.80 - 12.44 | 0.101 | 4.40 | 0.53 - 36.48 | 0.170 | 1.24 | 0.30 - 5.06 | 0.766 |
| Previous myocardial infarction or stroke | Yes (No) | 2.28 | 0.52 - 9.94 | 0.271 | ND | ND | ND | 1.36 | 0.43 - 4.33 | 0.603 |
| IDH wild-type | Yes (No) | 0.57 | 0.25 - 1.28 | 0.172 | 1.13 | 0.32 - 3.99 | 0.849 | 3.66 | 1.86 - 7.18 | <0.001 |
| Tumor size | 2.5 to 5 cm (< 2.5 cm) | 0.75 | 0.25 - 2.22 | 0.602 | 0.53 | 0.12 - 2.32 | 0.396 | 1.91 | 0.73 - 5.02 | 0.187 |
|  | ≥ 5 cm (< 2.5 cm) | 0.97 | 0.35 - 2.71 | 0.958 | 0.57 | 0.14 - 2.34 | 0.435 | 2.42 | 0.95 - 6.20 | 0.065 |
| Length of hospital days | ≥ 7 days (< 7 days) | 2.01 | 0.90 - 4.48 | 0.087 | 1.66 | 0.51 - 5.44 | 0.403 | 2.20 | 1.33 - 3.64 | 0.002 |
| Duration of prophylaxis | 3 months (1 month) | 0.97 | 0.03 - 27.20 | 0.985 | ND | ND | ND | ND | ND | ND |
|  | 6 months (1 month) | 0.43 | 0.02 - 11.86 | 0.618 | 0.84 | 0.10 - 7.58 | 0.873 | ND | ND | ND |
|  | During hospitalization (1 month) | 0.07 | 0.01 - 0.89 | 0.041 | 0.09 | 0.02 - 0.45 | 0.003 | ND | ND | ND |
|  | Others (1 month) | 0.14 | 0.01 - 3.22 | 0.218 | ND | ND | ND | ND | ND | ND |
| Hemiparesis/hemiplegia | Yes (No) | 1.42 | 0.71 - 2.81 | 0.318 | 7.67 | 1.66 - 35.47 | 0.009 | 2.35 | 1.47 - 3.76 | <0.001 |
| Immobilization | Yes (No) | 2.89 | 1.26 - 6.62 | 0.012 | 7.55 | 2.05 - 27.81 | 0.002 | 1.72 | 0.93 - 3.16 | 0.082 |
| Corticosteroid use | Yes (No) | 4.61 | 1.10 - 19.33 | 0.037 | ND | ND | ND | 2.73 | 1.25 - 5.95 | 0.012 |
| Antiplatelet use | Yes (No) | 2.43 | 0.84 - 7.04 | 0.102 | 2.18 | 0.28 - 17.18 | 0.461 | 0.26 | 0.04 - 1.85 | 0.177 |
| Radiotherapy | Yes (No) | 1.17 | 0.41 - 3.35 | 0.766 | 0.22 | 0.07 - 0.69 | 0.009 | 0.22 | 0.13 - 0.36 | <0.001 |
| Chemotherapy | Yes (No) | 1.60 | 0.56 - 4.58 | 0.377 | 0.29 | 0.10 - 0.89 | 0.030 | 0.26 | 0.16 - 0.43 | <0.001 |

ND: no data; cell counts were too low to perform statistical analyses.
