# Supplementary material for: Venous thromboembolism and secondary outcomes of bleeding and mortality in patients with gliomas: a multicenter cohort study
Source: Front Oncol. 2026 May 21;16:1771694. doi: 10.3389/fonc.2026.1771694 (PMC13233262; doi:10.3389/fonc.2026.1771694)
Supplement: Supplementary file 6 [file Table6.docx]

Supplementary Table 4 – Number and percentage of participants with CNS according to demographic, lifestyle, and health history characteristics.

| Variables | Categories | Total (334) | VTE (n=41) | Bleeding (n=14) | Death (n=96) |
| --- | --- | --- | --- | --- | --- |
|  |  | n (%) | n (%) | n (%) | n (%) |
| Sex | Male | 222 (66.5) | 29 (70.7) | 10 (71.4) | 68 (70.8) |
|  | Female | 112 (33.5) | 12 (29.3) | 4 (28.6) | 28 (29.2) |
| Age group | < 40 | 82 (24.6) | 7 (17.1) | 0 (0.0) | 5 (5.2) |
|  | 40 - 60 | 144 (43.1) | 19 (46.3) | 6 (42.9) | 46 (47.9) |
|  | > 60 | 108 (32.3) | 15 (36.6) | 8 (57.1) | 45 (46.9) |
| Education level | < Elementary school | 56 (16.8) | 7 (17.1) | 1 (7.1) | 25 (26.0) |
|  | Elementary school | 34 (10.2) | 3 (7.3) | 1 (7.1) | 10 (10.5) |
|  | High school | 78 (23.4) | 6 (14.6) | 4 (28.6) | 15 (15.6) |
|  | Higher education | 104 (31.1) | 16 (39.0) | 5 (35.7) | 25 (26.0) |
|  | Missing | 62 (18.6) | 9 (22.0) | 3 (21.4) | 21 (21.9) |
| Nutritional status | Underweight | 25 (7.5) | 4 (9.8) | 1 (7.1) | 8 (8.3) |
|  | Eutrophic | 110 (32.9) | 11 (26.8) | 2 (14.3) | 36 (37.5) |
|  | Overweight | 105 (31.4) | 16 (39.0) | 6 (42.9) | 27 (28.2) |
|  | Obese | 88 (26.3) | 10 (24.4) | 5 (35.7) | 25 (26.0) |
|  | Missing | 6 (1.8) | 0 (0.0) | 0 (0.0) | 0 (0.0) |
| Smoking | No | 237 (71) | 26 (63.4) | 10 (71.5) | 62 (64.6) |
|  | Yes | 65 (19.5) | 9 (22.0) | 3 (21.4) | 24 (25.0) |
|  | Missing | 32 (9.6) | 6 (14.6) | 1 (7.1) | 10 (10.4) |
| Alcoholism | No | 230 (68.9) | 29 (70.7) | 12 (85.7) | 64 (66.7) |
|  | Yes | 57 (17.1) | 5 (12.2) | 0 (0.0) | 19 (19.8) |
|  | Missing | 47 (14.1) | 7 (17.1) | 2 (14.3) | 13 (13.5) |
| Hypertension | No | 202 (60.5) | 19 (46.3) | 7 (50.0) | 50 (52.1) |
|  | Yes | 129 (38.6) | 21 (51.3) | 7 (50.0) | 45 (46.9) |
|  | Missing | 3 (0.9) | 1 (2.4) | 0 (0.0) | 1 (1.0) |
| Diabetes mellitus | No | 271 (81.1) | 27 (65.9) | 8 (57.1) | 76 (79.2) |
|  | Yes | 53 (15.9) | 13 (31.7) | 6 (42.9) | 14 (14.5) |
|  | Missing | 10 (3) | 1 (2.4) | 0 (0.0) | 6 (6.3) |
| Obesity | No | 233 (69.8) | 29 (70.7) | 8 (57.2) | 67 (69.8) |
|  | Yes | 79 (23.7) | 9 (22.0) | 5 (35.7) | 23 (24.0) |
|  | Missing | 22 (6.6) | 3 (7.3) | 1 (7.1) | 6 (6.2) |
| Chronic kidney failure | No | 303 (90.7) | 34 (82.9) | 8 (57.2) | 81 (84.4) |
|  | Yes | 2 (0.6) | 0 (0.0) | 1 (7.1) | 0 (0.0) |
|  | Missing | 29 (8.7) | 7 (17.1) | 5 (35.7) | 15 (15.6) |
| Congestive heart failure | No | 304 (91) | 34 (82.9) | 9 (64.3) | 83 (86.5) |
|  | Yes | 2 (0.6) | 0 (0.0) | 1 (7.1) | 0 (0.0) |
|  | Missing | 28 (8.4) | 7 (17.1) | 4 (28.6) | 13 (13.5) |
| Previous VTE | No | 269 (80.5) | 23 (56.1) | 9 (64.3) | 75 (78.1) |
|  | Yes | 16 (4.8) | 12 (29.3) | 0 (0.0) | 4 (4.2) |
|  | Missing | 49 (14.7) | 6 (14.6) | 5 (35.7) | 17 (17.7) |
| COPD | No | 300 (89.8) | 31 (75.6) | 10 (71.5) | 79 (82.3) |
|  | Yes | 9 (2.7) | 3 (7.3) | 1 (7.1) | 4 (4.2) |
|  | Missing | 25 (7.5) | 7 (17.1) | 3 (21.4) | 13 (13.5) |
| Previous myocardial infarction or stroke | No | 295 (88.3) | 32 (78.0) | 10 (71.4) | 80 (83.3) |
|  | Yes | 12 (3.6) | 2 (4.9) | 0 (0.0) | 3 (3.1) |
|  | Missing | 27 (8.1) | 7 (17.1) | 4 (28.6) | 13 (13.5) |

Previous VTE = Deep vein thrombosis or pulmonary embolism; COPD = Chronic obstructive pulmonary disease
