# Supplementary material for: Venous thromboembolism and secondary outcomes of bleeding and mortality in patients with gliomas: a multicenter cohort study
Source: Front Oncol. 2026 May 21;16:1771694. doi: 10.3389/fonc.2026.1771694 (PMC13233262; doi:10.3389/fonc.2026.1771694)
Supplement: Supplementary file 7 [file Table7.docx]

Supplementary Table 7 –Univariate Cox regression analyses for mortality in patients with GBM.

| Variables | Categories | Oligodendroglioma/ Astrocitoma | | | Gliomas NOS | | | GBM | | |
| --- | --- | --- | --- | --- | --- | --- | --- | --- | --- | --- |
|  |  | HR | 95% CI | p | HR | 95% CI | p | HR | 95% CI | p |
| Sex | female (male) | 0.02 | 0.00 – 1654.54 | 0.491 | 1.10 | 0.23 – 5.20 | 0.901 | 1.02 | 0.60 – 1.74 | 0.932 |
| Age group | 40 a 60 (< 40) | ND | ND | ND | 3.91 | 0.46 – 33.53 | 0.213 | 1.80 | 0.43 – 7.53 | 0.420 |
|  | > 60 (< 40) | ND | ND | ND | 5.49 | 0.61 – 49.19 | 0.128 | 1.99 | 0.47 – 8.31 | 0.348 |
| Education level | Elementary school (< Elementary) | ND | ND | ND | 0.70 | 0.14 – 3.48 | 0.664 | 0.97 | 0.46 – 2.04 | 0.931 |
|  | High school (< Elementary) | ND | ND | ND | 0.53 | 0.09 – 3.19 | 0.489 | 0.31 | 0.14 – 0.74 | ***0.008*** |
|  | Higher education (< Elementary) | ND | ND | ND | 0.39 | 0.07 – 2.32 | 0.298 | 0.73 | 0.41 – 1.30 | 0.279 |
| Nutritional status | Underweight (Eutrophic) | ND | ND | ND | ND | ND | ND | 0.79 | 0.33 – 1.93 | 0.609 |
|  | Overweight (Eutrophic) | ND | ND | ND | 0.19 | 0.02 – 1.72 | 0.140 | 0.62 | 0.34 – 1.13 | 0.119 |
|  | Obese (Eutrophic) | ND | ND | ND | 1.47 | 0.39 – 5.47 | 0.568 | 0.56 | 0.29 – 1.08 | 0.084 |
| Smoking | Yes (No) | ND | ND | ND | 1.18 | 0.31 – 4.57 | 0.809 | 1.19 | 0.68 – 2.07 | 0.545 |
| Alcoholism | Yes (No) | ND | ND | ND | 1.80 | 0.47 – 6.97 | 0.395 | 1.28 | 0.71 – 2.32 | 0.418 |
| Hypertension | Yes (No) | 2.31 | 0.15 – 37.00 | 0.553 | 2.84 | 0.82 – 9.85 | 0.101 | 0.85 | 0.52 – 1.40 | 0.534 |
| Diabetes mellitus | Yes (No) | 7.26 | 0.45 – 117. 30 | 0.163 | 1.97 | 0.42 – 9.32 | 0.391 | 0.49 | 0.24 – 1.00 | 0.051 |
| Obesity | Yes (No) | 2.35 | 0.15 – 37.55 | 0.546 | 1.73 | 0.45 – 6.73 | 0.429 | 0.86 | 0.49 – 1.49 | 0.586 |
| Chronic kidney failure | Yes (No) | ND | ND | ND | 6.70 | 1.35 – 33.37 | ***0.020*** | 1.01 | 0.46 – 2.22 | 0.983 |
| Congestive heart failure | Yes (No) | ND | ND | ND | 1.46 | 0.31 – 6.89 | 0.631 | 0.72 | 0.29 – 1.79 | 0.477 |
| Previous VTE | Yes (No) | ND | ND | ND | 1.31 | 0.28 – 6.16 | 0.736 | 0.80 | 0.42 – 1.54 | 0.512 |
| COPD | Yes (No) | ND | ND | ND | 1.46 | 0.31 – 6.89 | 0.631 | 0.96 | 0.44 – 2.12 | 0.929 |
| Previous myocardial infarction or stroke | Yes (No) | 12.80 | 0.80 – 204.90 | 0.071 | 2.64 | 0.56 – 12.49 | 0.220 | 0.67 | 0.29 – 1.56 | 0.355 |
| Tumor grade | 3 or 4 | 1.24 | 0.08 – 19.99 | 0.878 | 0.82 | 0.23 – 2.92 | 0.765 | ND | ND | ND |
| IDH wild-type | Yes (No) | ND | ND | ND | 1.32 | 0.37 – 4.70 | 0.664 | 1.45 | 0.63 – 3.37 | 0.386 |
| Tumor size | 2.5 to 5 cm (< 2.5 cm) | ND | ND | ND | 3.99 | 0.47 – 34.25 | 0.207 | 0.69 | 0.33 – 1.47 | 0.334 |
|  | ≥ 5 cm (< 2.5 cm) | ND | ND | ND | 1.88 | 0.21 – 16.87 | 0.571 | 1.25 | 0.61 – 2.55 | 0.539 |
| Length of hospital days | ≥ 7 days (< 7 days) | ND | ND | ND | 9.16 | 1.92 – 43.71 | ***0.005*** | 1.45 | 0.88 – 2.37 | 0.142 |
| Hemiparesis/hemiplegia | Yes (No) | ND | ND | ND | 2.62 | 0.74 – 9.29 | 0.136 | 1.18 | 0.72 – 1.93 | 0.517 |
| Immobilization | Yes (No) | 8.83 | 0.55 – 142.88 | 0.125 | 3.49 | 1.01 – 12.11 | 0.048 | 0.81 | 0.46 – 1.42 | 0.456 |
| Corticosteroid use | Yes (No) | ND | ND | ND | ND | ND | ND | 1.12 | 0.57 – 2.20 | 0.745 |
| Antiplatelet use | Yes (No) | ND | ND | ND | 0.04 | 0.00 – 50.64 | 0.372 | 1.24 | 0.68 – 2.24 | 0.485 |
| Radiotherapy | Yes (No) | 0.12 | 0.01 – 1.94 | 0.135 | 0.08 | 0.02 – 0.31 | ***<0.001*** | 0.13 | 0.07 – 0.23 | ***<0.001*** |
| Chemotherapy | Yes (No) | ND | ND | ND | 0.07 | 0.02 – 0.30 | ***<0.001*** | 0.16 | 0.09 – 0.29 | ***<0.001*** |
| VTE after surgery | Yes (No) | ND | ND | ND | 2.94 | 0.37 – 23.39 | 0.307 | 0.61 | 0.29 – 1.29 | 0.195 |
| Bleeding after surgery | Yes (No) | ND | ND | ND | ND | ND | ND | 2.58 | 1.11 – 6.01 | ***0.027*** |
| ND: no data; cell counts were too low to perform statistical analyses; CD: correlated data; NA: not applicable (the outcome occurred prior to the event of interest). | | | | | | | | | | |
