# Supplementary material for: Venous thromboembolism and secondary outcomes of bleeding and mortality in patients with gliomas: a multicenter cohort study
Source: Front Oncol. 2026 May 21;16:1771694. doi: 10.3389/fonc.2026.1771694 (PMC13233262; doi:10.3389/fonc.2026.1771694)
Supplement: Supplementary file 8 [file Table8.docx]

Supplementary Table 8 – Univariate Cox regression analyses for mortality in patients with grade 3 or 4 tumors.

| Variables | Categories | G2 | | | G3 or 4 | | |
| --- | --- | --- | --- | --- | --- | --- | --- |
|  |  | HR | 95% CI | p | HR | 95% CI | p |
| Sex | female (male) | 0.02 | 0.00 – 44.20 | 0.329 | 0.93 | 0.56 – 1.55 | 0.793 |
| Age group | 40 - 60 (< 40) | ND | ND | ND | 5.53 | 1.70 – 18.04 | ***0.005*** |
|  | > 60 (< 40) | ND | ND | ND | 6.96 | 2.14 – 22.68 | ***0.001*** |
| Education level | Elementary school (< Elementary) | 1.66 | 0.15 – 18.28 | 0.680 | 0.91 | 0.46 – 1.80 | 0.779 |
|  | High school (< Elementary) | 0.75 | 0.07 – 8.32 | 0.818 | 0.24 | 0.10 – 0.54 | ***0.001*** |
|  | Higher education (< Elementary) | ND | ND | ND | 0.61 | 0.35 – 1.06 | 0.077 |
| Nutritional status | Underweight (Eutrophic) | ND | ND | ND | 0.74 | 0.33 – 1.69 | 0.477 |
|  | Overweight (Eutrophic) | 2.11 | 0.19 – 23.28 | 0.542 | 0.54 | 0.30 – 0.98 | ***0.041*** |
|  | Obese (Eutrophic) | 3.77 | 0.34 – 41.57 | 0.279 | 0.53 | 0.29 – 0.97 | ***0.040*** |
| Smoking | Yes (No) | 1.16 | 0.13 – 10.42 | 0.892 | 1.14 | 0.67 – 1.93 | 0.630 |
| Alcoholism | Yes (No) | 2.36 | 0.39 – 14.12 | 0.348 | 1.18 | 0.67 – 2.09 | 0.569 |
| Hypertension | Yes (No) | 4.82 | 0.81 – 28.87 | 0.085 | 1.14 | 0.71 – 1.83 | 0.584 |
| Diabetes mellitus | Yes (No) | 5.88 | 0.98 – 35.34 | 0.053 | 0.67 | 0.34 – 1.30 | 0.236 |
| Obesity | Yes (No) | 4.44 | 0.74 – 26.70 | 0.103 | 0.85 | 0.50 – 1.46 | 0.565 |
| Chronic kidney failure | Yes (No) | 4.68 | 0.52 – 42.23 | 0.169 | 1.16 | 0.55 -2.41 | 0.701 |
| Congestive heart failure | Yes (No) | 9.63 | 1.07 – 86.76 | 0.043 | 0.77 | 0.35 – 1.79 | 0.549 |
| Previous VTE | Yes (No) | 2.41 | 0.27 – 21.60 | 0.431 | 0.78 | 0.42 – 1.45 | 0.432 |
| COPD | Yes (No) | 6.04 | 0.67 – 54.13 | 0.108 | 0.93 | 0.45 – 1.95 | 0.854 |
| Previous myocardial infarction or stroke | Yes (No) | ND | ND | ND | 1.03 | 0.51 – 2.07 | 0.935 |
| Tumor type | Gliomas NOS (Oligodendroglioma/ Astrocitoma*) | 13.58 | 1.52 – 121.65 | 0.020 | 10.51 | 1.27 – 87.35 | ***0.029*** |
|  | GBM (Oligodendroglioma/ Astrocitoma*) | ND | ND | ND | 20.38 | 2.83 – 146.95 | ***0.003*** |
|  | Other (Oligodendroglioma/ Astrocitoma*) | ND | ND | ND | ND | ND | ND |
| IDH wild-type | Yes (No) | 2.78 | 0.47 – 16.68 | 0.262 | 2.57 | 1.31 – 5.02 | ***0.006*** |
| Tumor size | 2.5 to 5 cm (< 2.5 cm) | ND | ND | ND | 1.03 | 0.51 – 2.89 | 0.941 |
|  | ≥ 5 cm (< 2.5 cm) | ND | ND | ND | 1.36 | 0.69 – 2.68 | 0.378 |
| Length of hospital days | ≥ 7 days (< 7 days) | ND | ND | ND | 1.66 | 1.04 – 2.65 | ***0.035*** |
| Hemiparesis/hemiplegia | Yes (No) | 7.46 | 1.24 – 44.75 | ***0.028*** | 1.56 | 0.98 – 2.50 | 0.063 |
| Immobilization | Yes (No) | 10.52 | 1.76 – 63.04 | ***0.010*** | 1.10 | 0.65 – 1.86 | 0.733 |
| Corticosteroid use | Yes (No) | ND | ND | ND | 1.79 | 0.91 – 3.49 | 0.090 |
| Antiplatelet use | Yes (No) | ND | ND | ND | 1.04 | 0.58 – 1.87 | 0.892 |
| Radiotherapy | Yes (No) | 0.06 | 0.01 – 0.53 | ***0.011*** | 0.20 | 0.12 – 0.33 | ***<0.001*** |
| Chemotherapy | Yes (No) | 0.13 | 0.02 – 1.19 | 0.071 | 0.17 | 0.10 – 0.28 | ***<0.001*** |
| VTE after surgery | Yes (No) | ND | ND | ND | 0.75 | 0.37 – 1.50 | 0.413 |
| Bleeding after surgery | Yes (No) | 10.99 | 1.21 – 99.59 | ***0.033*** | 2.52 | 1.09 – 5.83 | ***0.031*** |
| ND: no data; cell counts were too low to perform statistical analyses; CD: correlated data; NA: not applicable (the outcome occurred prior to the event of interest). | | | | | | | |
